# Stepwise deprotonation of truxene: structures, metal complexation, and charge-dependent optical properties†

**DOI:** 10.1039/d3sc04885c

**Published:** 2023-10-31

**Authors:** Yumeng Guo, Herdya S. Torchon, Yikun Zhu, Zheng Wei, Zhenyi Zhang, Haixiang Han, Marina A. Petrukhina, Zheng Zhou

**Affiliations:** a School of Materials Science and Engineering, Tongji University Shanghai 201804 China zhouzheng@tongji.edu.cn; b Department of Chemistry, University at Albany, State University of New York Albany NY 12222 USA mpetrukhina@albany.edu; c Bruker (Beijing) Scientific Technology Co., Ltd Shanghai 200233 China

## Abstract

As a planar subunit of C_60_-fullerene, truxene (C_27_H_18_) represents a highly symmetrical rigid hydrocarbon with strong blue emission. Herein, we used truxene as a model to investigate the chemical reactivity of a fullerene fragment with alkali metals. Monoanion, dianion, and trianion products with different alkali metal counterions were crystallized and fully characterized, revealing the core curvature dependence on charge and alkali metal coordination. Moreover, a ^1^proton nuclear magnetic resonance study coupled with computational analysis demonstrated that deprotonation of the aliphatic CH_2_ segments introduces aromaticity in the five-membered rings. Importantly, the UV-vis absorption and photoluminescence of truxenyl anions with different charges reveal intriguing charge-dependent optical properties, implying variation of the electronic structure based on the deprotonation process. An increase in aromaticity and π-conjugation yielded a red shift in the absorption and photoluminescent spectra; in particular, large Stokes shifts were observed in the truxenyl monoanion and dianion with high emission quantum yield and time of decay. Overall, stepwise deprotonation of truxene provides the first crystallographically characterized examples of truxenyl anions with three different charges and charge-dependent optical properties, pointing to their potential applications in carbon-based functional materials.

## Abstract 2

Stepwise deprotonation of truxene with alkali metals affords truxenyl anions with different charges, exhibiting core curvature dependence on charge and metal binding. UV-vis and PL studies reveal charge-dependent optical properties, which is further supported by DFT calculations.

## Graphical abstract

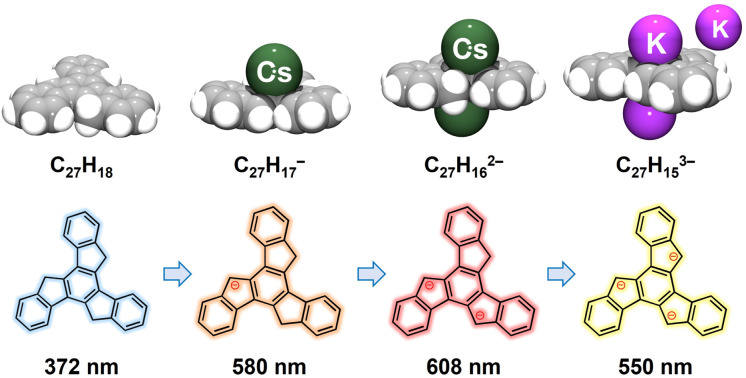

## Introduction

**Scheme 1:**
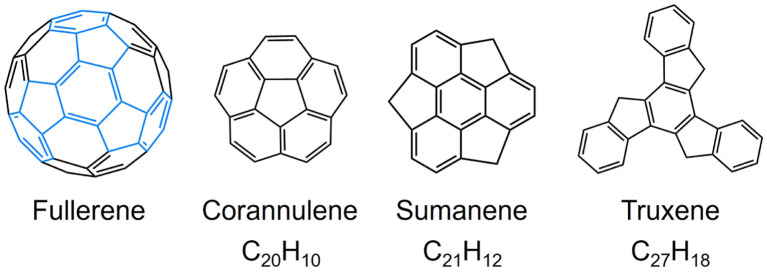
Depictions of fullerene and its subunits.

Since the discovery of C_60_-fullerene in 1985,^1^ molecular nanocarbons have attracted enormous attention in fundamental chemistry and materials science. Over the decades, a wide variety of molecular nanocarbon architectures with different sizes and topologies have been synthesized with tunable chemical and physical properties,^2–9^ which greatly expands their applications in energy storage, optoelectronic devices, and superconductors.^10–12^ In particular, the fusion of five-membered rings into the hexagonal carbon framework can cause changes in structure and reactivity.^13–18^ Taking some fullerene subunits as examples (Scheme 1), the central five-membered ring in corannulene generates a Gaussian curvature with *C*_5v_ symmetry, offering two distinctive π-surfaces (concave and convex) for metal binding. Corannulene (C_20_H_10_) exhibits a very rich redox chemistry and readily accepts up to four electrons upon chemical reduction, affording anionic species with interesting metal coordination or supramolecular chemistry.^19–21^ In contrast, sumanene (C_21_H_12_), built with three external five-membered rings, possesses a positive curvature (*C*_3v_ symmetry) with site-selective reactivity,^22,23^ and can undergo stepwise deprotonation to form either double-concave or double-convex metal complexes.^24,25^

Unlike bowl-shaped fragments, truxene (1, C_27_H_18_) can be considered a planar subunit of fullerene with *C*_3h_ molecular symmetry.^26,27^ However, the external six-membered rings can be readily functionalized to generate star-shaped molecules and oligomers.^28–33^ Moreover, the presence of three sp^3^-hybridized carbon atoms on the pentagonal rings imparts additional chemical reactivity for adding alkyl chains or heteroatoms.^34,35^ Notably, truxene exhibits strong blue emission due to its large optical gap.^36^ The combination of high symmetry, rigid planar structure, site-specific reactivity, and unique optical properties make the truxene unit a highly attractive building block for the construction of diverse novel materials with broad applications, such as organic photovoltaics, liquid crystals, and nonlinear optical materials.^28,29,33^

Adding charges into π-conjugated systems can cause variations in chemical and electronic structures, which further allows the modulation of their optical properties. Although truxene has many promising applications, its charge-dependent optical behavior has never been explored. We have employed the chemical reduction approach to prepare charged π-conjugated carbanions whose molecular structures were successfully established by X-ray single crystal diffraction.^21,37^ These studies demonstrated their structural transformations, thus opening the way for investigations into the optical, electronic, and magnetic properties of the resulting solid crystalline materials.^38–41^ Inspired by truxene's site-specific reactivity and interesting optical properties, we report the first comprehensive study of the stepwise deprotonation of truxene with alkali metals (Na, K, and Cs). Products in different charging states have been isolated and fully characterized by single crystal X-ray diffraction, revealing the core curvature dependence on the charge and original metal complexation. Furthermore, it was revealed that the truxenyl anions demonstrate charge-dependent absorption and photoluminescence properties. Therefore, density functional theory (DFT) was performed to gain deep insights into such optical behavior. This work represents the first structural-based charge-dependent study of truxene and shows its promising applications in carbon-based functional molecular materials.

## Results and discussion

### Solid state structure of 1

**Fig. 1:**
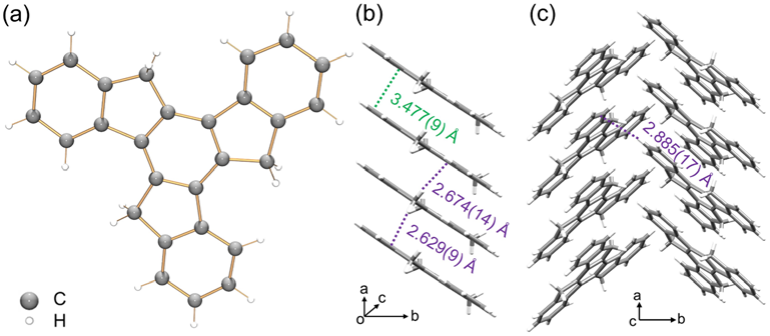
(a) Crystal structure of 1 as a ball-and-stick model, (b) 1D column and (c) solid-state structure as capped stick models with depictions of C–H⋯π (purple) and π⋯π (green) interactions.

To understand the structure–property correlation of the truxenyl framework, it is necessary to obtain the crystal structure of neutral truxene first and use it for the comparison with the reduced species. Although the synthesis of 1 was reported decades ago, its solid state structure remains unclear due to its highly disordered nature. In recent work by Zou *et al.*,^42^ truxene crystals were grown from a mixture of trichlorobenzene and methanol, but no further details were provided. Prior to a chemical reactivity study, 1 was purified by sublimation in a glass ampule *in vacuo* at elevated temperature (265 °C), affording nicely grown yellow needles in good yield (75%). The structural analysis shows 1 crystallizes in a *P*2_1_ space group (Fig. 1a). This indicates that the molecular assembly of truxene can be chiral; however, the molecules from two orientations are overlapped and cocrystallized due to the high disorder (Fig. S23†). In the solid state structure, the molecules of 1 are stacked into a 1D column with a deck-to-deck separation of 3.477(9) Å and a slip distance of 3.142(9) Å (Fig. 1b). Unlike the fully aligned stacks of carbon bowls,^22^ this slipped herringbone packing is commonly seen in most planar polyarenes.^43,44^ It should be noted that H-atoms on the sp^3^ carbons weaken the π⋯π interactions in 1 (3.477(9) Å); thus, the columnar packing is dominated by C–H⋯π interactions (2.629(9)–2.674(14) Å). The 1D columns are further linked into a 2D layer through a weaker C–H⋯π contact (2.885(17) Å, Fig. 1c).

### Stepwise deprotonation of 1

**Scheme 2:**
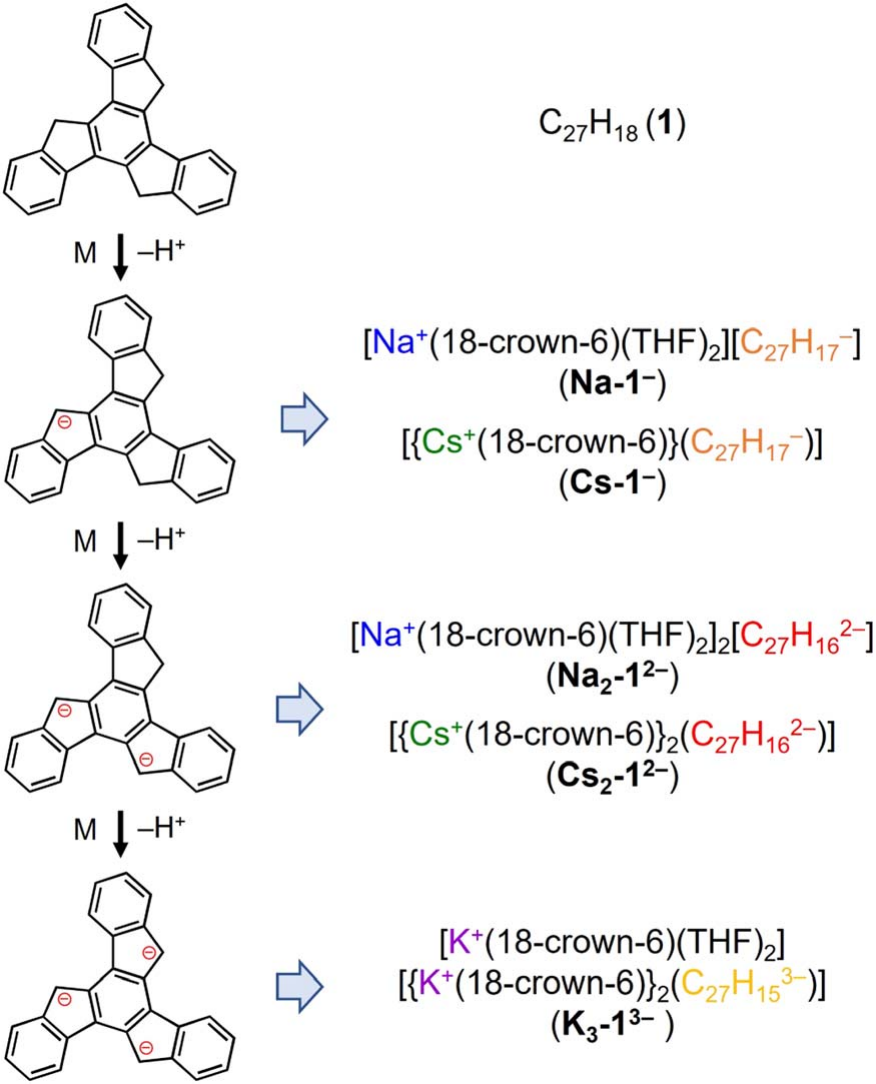
Preparation of truxenyl mono-, di-, and trianionic products with Na^+^, K^+^, and Cs^+^ ions.

Truxene has a rigid carbon framework with three “exposed” five-membered rings on the edge of the molecule, so multiple deprotonations could occur to afford truxenyl anions with different charges, similar to that of π-bowl sumanene.^23^ Additionally, its three-fold planar structure provides more chemically-equivalent sites for metal binding, which helps stabilize the high charging states in contrast to sumanene. The chemical reactivity of truxene (C_27_H_18_, 1) was investigated in tetrahydrofuran (THF) at room temperature with Na, K, and Cs metals. The reaction first proceeds through a bright orange color, which corresponds to the monoanionic stage, followed by an orange–red color (the dianion), and finally lightens to yellow, which indicates the formation of a truxenyl trianion. By controlling the reaction time of the alkali-metal-induced deprotonation, orange, red, and yellow plate-shaped crystals corresponding to different reaction stages have been successfully isolated and characterized by single crystal X-ray diffraction (Scheme 2, see ESI† for more details). An X-ray diffraction study confirmed the formation of two solvent-separated ion pairs with Na^+^ counterions, [Na^+^(18-crown-6)(THF)_2_][C_27_H_17_^−^] (Na-1^−^) and [Na^+^(18-crown-6)(THF)_2_]_2_[C_27_H_16_^2−^] (Na_2_-1^2−^, crystallized with four interstitial THF), and three contact–ion complexes, [{Cs^+^(18-crown-6)}(C_27_H_17_^−^)] (Cs-1^−^), [{Cs^+^(18-crown-6)}_2_(C_27_H_16_^2−^)] (Cs_2_-1^2−^), and [K^+^(18-crown-6)(THF)_2_][{K^+^(18-crown-6)}_2_(C_27_H_15_^3−^)] (K_3_-1^3−^, crystallized with one interstitial THF).

### ^1^H NMR investigation of truxenyl anions

**Fig. 2:**
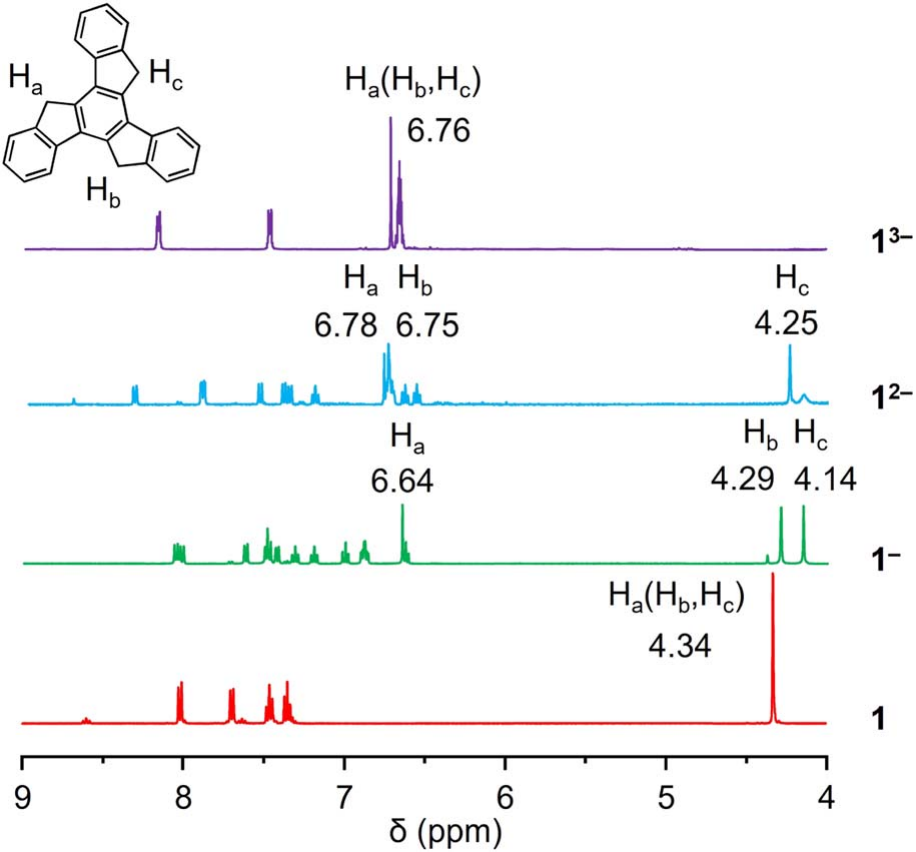
^1^H NMR spectra of 1, Na-1^−^, Na_2_-1^2−^, and K_3_-1^3−^ in THF-*d*_8_ at 25 °C.

A ^1^proton nuclear magnetic resonance (^1^H NMR) spectroscopic investigation was initially performed to understand the solution behavior of all the compounds (Fig. 2 and S1–S8†). In neutral 1, the singlet appearing at 4.34 ppm represents the H-atoms on the sp^3^-hybridized carbon atoms. The first deprotonation of one five-membered ring (loss of one H_a_) in Na-1^−^ affords an increase in aromaticity of the corresponding ring. As a result, the remaining H_a_ shifted largely downfield to the aromatic region (6.64 ppm), leaving the other two sets of aliphatic protons as singlets at 4.14 and 4.29 ppm (H_b_ and H_c_). The same splitting was observed in the truxene monoanion complexed with a Cs^+^ counterion in Cs-1^−^ (Fig. S4†). Unlike bowl-shaped sumanene, whose positive curvature enlarged the difference between the *endo*- and *exo*-protons,^22^ the peak H_b_, which stayed close to the high electron density area, also experienced deshielding and was slightly shifted downfield (4.37 ppm *vs.* 4.23 ppm for H_c_). Next, deprotonation occurs on the second five-membered ring to afford truxenyl dianions in Na_2_-1^2−^ and Cs_2_-1^2−^ (Fig. S5†) accompanied by the loss of one H_b_ and deshielding of the other. Consequently, both remaining H_a_ and H_b_ were greatly deshielded and shifted to the aromatic region, while H_c_ remained in the aliphatic region. Notably, the singlet for H_c_ confirmed the similarity of the protons on both sides and the planarity of the carbon framework. Finally, in the fully deprotonated trianion in K_3_-1^3−^, the proton signals appear as a singlet observed at 6.76 ppm due to its highly symmetrical structure.

### Crystallographic analysis of truxenyl anions

**Fig. 3:**
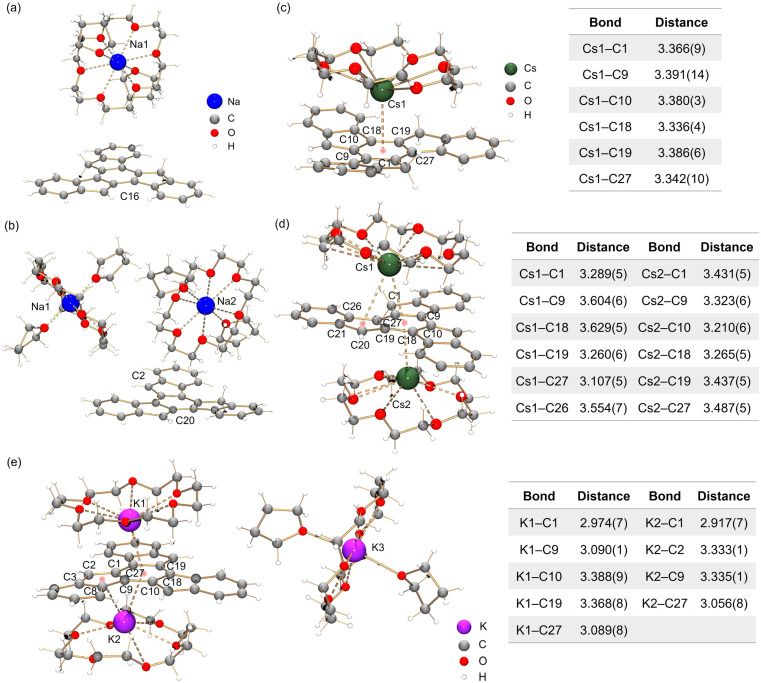
Crystal structures of (a) Na-1^−^, (b) Na_2_-1^2−^, (c) Cs-1^−^, (d) Cs_2_-1^2−^ and (e) K_3_-1^3−^ as ball-and-stick models, along with tables of Cs–C and K–C bond distances (Å).

In the crystal structure of Na-1^−^ (Fig. 3a), a single deprotonation occurs at the C16 atom and the {Na^+^(18-crown-6)(THF)_2_} moiety is solvent-separated from the C_27_H_17_^−^ core. Interestingly, in contrast to sumanene where only one-fold deprotonation was observed with Na metal,^25^ extending the reaction time allows the formation of a “naked” C_27_H_16_^2−^ dianion with two Na^+^ counterions (Fig. 3b, deprotonation at C2 and C20). In both structures, each Na^+^ ion is wrapped with 18-crown-6 ether and capped by two THF molecules, with the Na–O distances (Na–O_crown_: 2.575(12)–2.839(11) Å, Na–O_THF_: 2.301(10)–2.350(12) Å) being comparable to the values reported in the literature.^38,45–47^

By using Cs metal, the reaction undergoes the same color changes but gives rise to two contact–ion complexes. In the crystal structure of Cs-1^−^ (Fig. 3c), the Cs^+^ ion is coordinated to the central six-membered ring of C_27_H_17_^−^ in an η^6^-manner, with the Cs–C distances ranging over 3.336(4)–3.391(14) Å. In the crystal structure of Cs_2_-1^2−^ (Fig. 3d), there are two independent Cs^+^ ions coordinated to the truxenyl dianion, C_27_H_16_^2−^. The Cs1 ion is bound to the central six-membered ring in an asymmetric *η*^6^-fashion, with the Cs–C distances over spanning a broad range of 3.107(8)–3.629(8) Å. Additional contact is found between Cs1 and the adjacent five-membered ring E (3.554(8) Å). The Cs2 ion is only bound to the central six-membered ring in an η^6^-mode (3.210(8)–3.487(8) Å). All Cs–C distances are close but shorter than the previously reported values for bowl-shaped anions.^25,48^ In both structures, each Cs^+^ ion is also asymmetrically bound to an 18-crown-6 ether molecule, with Cs–O distances of 2.983(18)–3.211(18) Å.

In contrast to the sumanenyl supramolecular aggregate with K^+^ counterions formed by a mixture of dianions and trianions,^24^ the reaction of truxene with K metal yields a pure trianionic product. In the crystal structure of K_3_-1^3−^ (Fig. 3e), two K^+^ ions are bound to the truxenyl trianion and entrapped by an 18-crown-6 ether molecule, with the K–C distances ranging from 3.107(8) Å to 3.629(8) Å. Unlike Cs ions in Cs_2_-1^2−^, the K1 ion in K_3_-1^3−^ sits closer to the C_27_H_15_^3−^ core (2.909(8) Å), but the K2 ion is slipped over two rings (3.122(8)/3.301(8) Å). The remaining K^+^ ion is fully wrapped by 18-crown-6 ether and capped by two THF molecules.

The structural perturbation of the truxenyl core upon stepwise deprotonation can be illustrated by dihedral angles and C–C bond distances compared with the neutral parent (scheme in Tables 1 and S2†). Notably, one-fold deprotonation of 1 leads to an increase in aromaticity of the deprotonated five-membered ring E in Na-1^−^ and Cs-1^−^. The C–C bonds around the deprotonated carbon atom (a, b) are significantly shortened from 1.500(10) Å in C_27_H_18_ to 1.443(14) Å in Na-1^−^ and 1.429(6) Å in Cs-1^−^. Additional bond length reduction is also observed in the two-fold deprotonated C_27_H_16_^2−^ (c, d in Na_2_-1^2−^ and Cs_2_-1^2−^) and three-fold deprotonated C_27_H_15_^3−^ (c, d, e, and f in K_3_-1^3−^). Moreover, C–C bonds around the central ring R (g, h, and i) in all charged species show a slight increase in length compared to 1 (*e.g.*, 1.420(7) Å in Na_2_-1^2−^*vs.* 1.400(7) Å in 1). In addition, the truxenyl anions experience some curvature after deprotonation, as shown by an increase in dihedral angles around the central six-membered ring (Table 1). In neutral 1, the truxene core is planar. A small curvature is observed upon one-fold deprotonation in Na-1^−^ (2.9°). It should be noted that the direct Cs^+^ ion coordination in Cs-1^−^ yields a more pronounced curvature for the truxenyl monoanion (9.9°), forming a bowl-shaped framework with a bowl depth of 0.606(6) Å. After two-fold deprotonation, the C_27_H_16_^2−^ core in Na_2_-1^2−^ becomes more twisted (7.9°) while that effect is less pronounced in Cs_2_-1^2−^, probably due to double-sided metal binding. Finally, stemming from increased aromaticity and direct metal coordination, a curvature reduction is observed in the C_27_H_15_^3−^ core in K_3_-1^3−^ (4.1°).

**Table 1:** Geometric comparison in truxenyl cores along with a labeling scheme

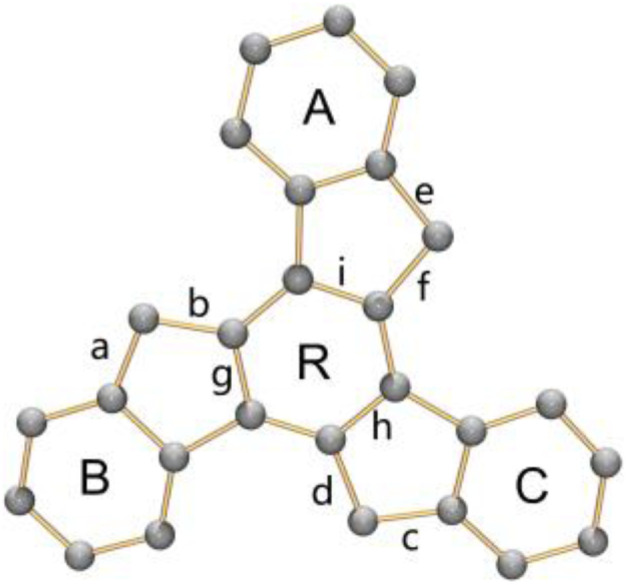						
Dihedral angle (°)	1	Na-1^−^	Na_2_-1^2−^	Cs-1^−^	Cs_2_-1^2−^	K_3_-1^3−^
A/R	0	2.4	10.5	15.0	5.4	2.1
B/R	0	4.2	3.3	8.9	9.4	4.0
C/R	0	2.2	9.8	5.8	5.6	6.2
Avg.	0	2.9	7.9	9.9	6.8	4.1

### Aromaticity evaluation of truxenyl anions

**Fig. 4:**
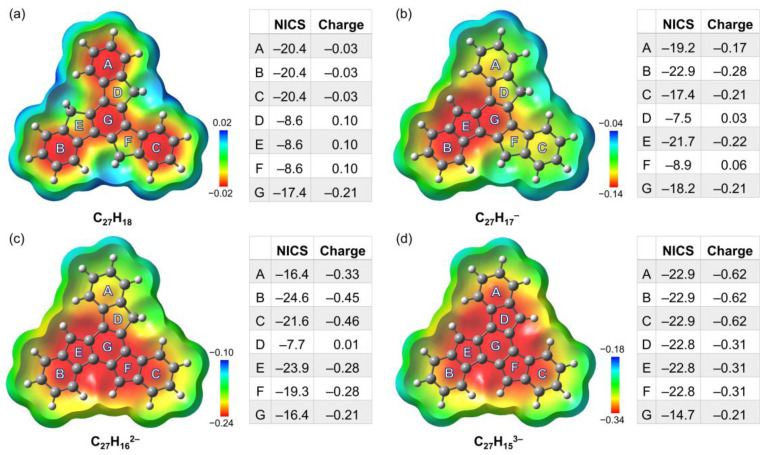
Electrostatic potential maps (mapped on the electron density using an isovalue of 0.0004 and Z-clip of 0.4) of (a) C_27_H_18_, (b) C_27_H_17_^−^, (c) C_27_H_16_^2−^, and (d) C_27_H_15_^3−^, along with tables of the corresponding NICS(1.7)_ZZ_ values (ppm) and Lowdin partial charges for each ring.

To better understand the geometric, aromatic, and electronic changes of the parent truxene (1) compared to its singly-, doubly-, and triply-deprotonated products, density functional theory (DFT) calculations were performed at the B3LYP-D3BJ/def2-TZVP level. Stanger *et al.* previously showed that the NICS(1.7)_ZZ_ value is sufficient to eliminate the disturbance generated by σ-electrons when describing the aromaticity in polycyclic aromatic hydrocarbon (PAH) systems and their charged species;^49^ thus, it is used below for discussion and comparison. The fully optimized gas-phase neutral molecule 1 adopts a planar structure (Fig. 4a), which is similar to the geometry observed in the crystal structure. The NICS(1.7)_ZZ_ values of four six-membered rings (A, B, C, and G) indicate that each of them has an aromatic character (avg. −19.6 ppm, or 89% of benzene; NICS(1.7)_ZZ_ = −22 ppm for benzene at the same level of theory). In contrast, three five-membered rings D, E, and F are noticeably less aromatic. Due to the high symmetry of the molecule (*D*_3h_), no difference exists between rings A, B, and C as well as rings D, E, and F.

After the first deprotonation, the monoanion (C_27_H_17_^−^) remains planar, but the symmetry is reduced to *C*_s_ (Fig. 4b); an increase in aromaticity is observed, particularly around rings B, E, and G. Notably, the loss of a proton on ring E yields more pronounced aromaticity (−21.7 ppm), which is close to 95% that of benzene, similar to a previous investigation of sumanene, a bowl-shaped subunit of C_60_-fullerene.^24^

Following the second deprotonation to afford the “naked” dianion (Fig. 4c), the aromaticity of the system further increases. Notably, the NICS(1.7)_ZZ_ values of rings B and E (−24.6/−23.9 ppm, 112%/108% of benzene, respectively) are more pronounced than those of rings C and F (−21.6/−19.3 ppm, 98%/88% of benzene, respectively). This can be further identified by the large difference in their partial charges (avg. −0.46 for B/C *vs.* −0.28 for E/F), which indicates an asymmetric charge distribution that could further affect metal coordination upon complexation. In contrast, the NICS(1.7)_ZZ_ value of outer ring A next to ring D further reduces to −16.4 ppm.

Upon further deprotonation (Fig. 4d), the aromaticity of the trianion further increases. Apart from central ring G, all the rings have a very high NICS(1.7)_ZZ_ of −22.8 pm (103% of benzene). The reduced NICS value for central ring G is consistent with the observation for three-fold deprotonated sumanene, where the individual contribution of the ring becomes less prominent when involved in total delocalization.^50^ The truxenyl trianion is reverted to *D*_3h_ symmetry, and the electrons are fully delocalized.

**Fig. 5:**
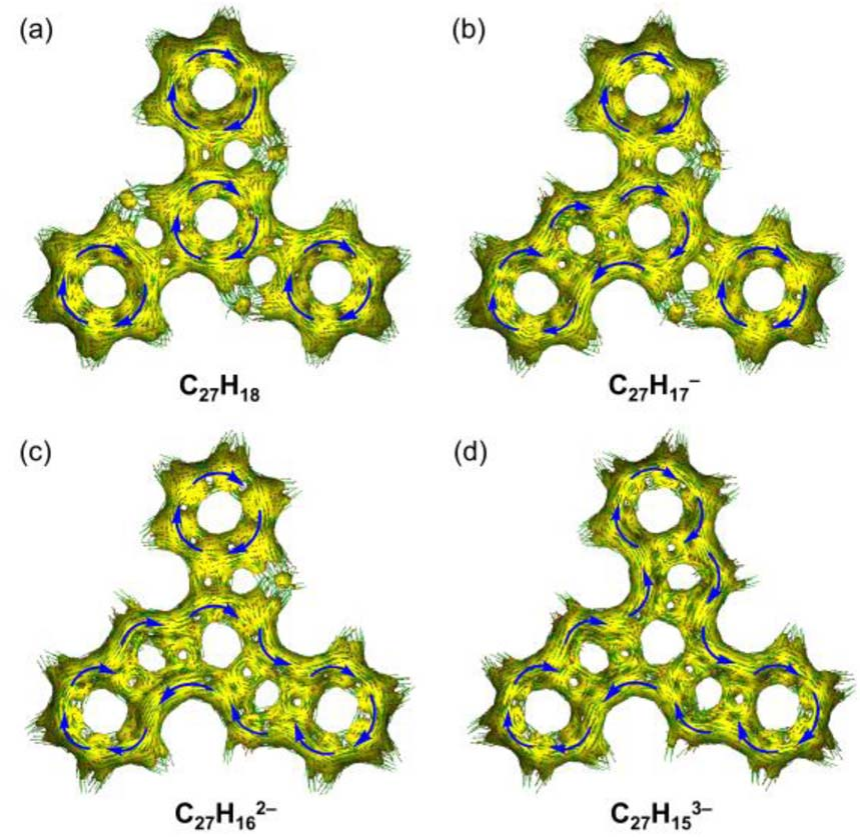
Full ACID isosurfaces for (a) C_27_H_18_, (b) C_27_H_17_^−^, (c) C_27_H_16_^2−^, and (d) C_27_H_15_^3−^. Current density vectors are plotted onto the ACID isosurface to indicate diatropic (blue) ring currents.

The change in aromaticity can also be observed from the calculated anisotropy of current-induced density (ACID) plots for neutral truxene and the respective anions (Fig. 5 and S40†). In neutral 1, the presence of three protonated pentagonal rings breaks the conjugation between adjacent six-membered rings to a certain extent, enabling each of them to sustain local ring currents. Thus, four separate diatropic ring current centers are found in the ACID plot of 1 (Fig. 5a), which is more evident without σ-orbitals (Fig. S40a†). After one-fold deprotonation, one five-membered ring becomes aromatic and serves as a bridge that connects the adjacent two six-membered rings. As a result, a larger diatropic ring current is formed along one arm of the truxene monoanion (Fig. 5b and S40b†). With the increase in negative charge on the carbon framework, the second and third five-membered rings lose their proton and become aromatic, yielding a trianionic product with charge delocalization over the whole molecular core (Fig. 5c, d, S40c and d†). It is also concluded that the deprotonation of three five-membered rings changes the ring current, yielding a diatropic ring current around the periphery.

### Charge-dependent optical property of truxenyl anions

As shown above, the variation in charges achieved by controlled stepwise deprotonation provides a series of truxenyl anions with charging states ranging from 0 to −3, which possess entirely different electronic structures that further affect their optical properties. Based on the ^1^H NMR and UV-vis spectroscopic analyses, the Na- and Cs-complexed products behave similarly in solution, so Na-1^−^, Na_2_-1^2−^, and K_3_-1^3−^ were selected as representatives for truxenyl monoanion, dianion, and trianion and used for comparison with the neutral parent.

**Fig. 6:**
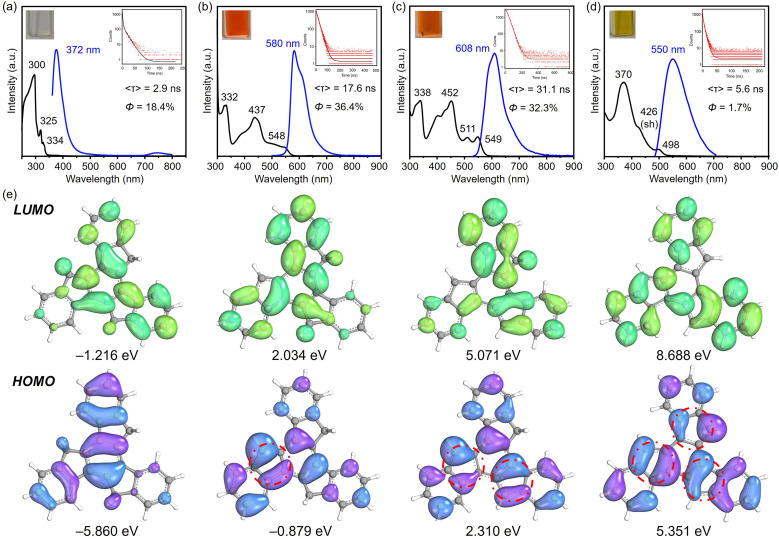
UV-vis absorption (black) and photoluminescence emission spectra (blue, excited at 350 nm) of (a) 1, (b) Na-1^−^, (c) Na_2_-1^2−^, and (d) K_3_-1^3−^ in THF at 25 °C. (e) Electron density distribution of the frontier molecular orbitals (isovalue = 0.02) of C_27_H_18_, C_27_H_17_^−^, C_27_H_16_^2−^, and C_27_H_15_^3−^ (B3LYP-D3BJ/def2-TZVP/CPCM(THF)).

First, UV-vis spectroscopy assisted by TD-DFT calculation was used to visualize the absorption properties and natural transitions (see ESI† for more details). In general, the nearly planar truxenyl framework is dominated by the π → π* transition in neutral and in charged states. The major absorption (*λ*_abs_) of 1 dissolved in THF occurs at 300 nm with a large energy gap (Fig. 6a). Upon deprotonation, Na-1^−^ shows intense bands with an absorption maximum at 332 nm (Fig. 6b), which is correlated with the HOMO−1 → LUMO electronic transition (oscillator strength *f* = 0.524) according to the TD-DFT calculation, while the lowest-energy absorption band (*λ*_abs_ = 548 nm) comes from the HOMO → LUMO electronic transition (*f* = 0.205). In contrast, the largest absorption band in Na_2_-1^2−^ is found at 338 nm (Fig. 6c, HOMO−1 → LUMO+3, *f* = 1.165), while the low-energy bands in accordance with the HOMO−1 → LUMO and HOMO → LUMO electronic transitions (*f* = 0.160/0.048) are found at 511 and 549 nm, respectively. Finally, K_3_-1^3−^ shows an absorption maximum at 370 nm (Fig. 6d), originating mainly from the HOMO−2 → LUMO and HOMO−2 → LUMO+1 electronic transitions (*f* = 1.009/1.011). As shown in Fig. 6e, the HOMO → LUMO electron transitions in Na-1^−^, Na_2_-1^2−^, and K_3_-1^3−^ occur from the deprotonated rings (bonding π-orbitals) to the protonated rings (antibonding π*-orbitals), from which the increasing π-conjugation yields a red shift in their absorption spectra.

Next, to understand the light-emitting behavior of the truxenyl framework upon stepwise deprotonation, photoluminescence spectroscopy was performed. Truxene is a good monomer for building fluorescent materials with blue emission.^14^ In neutral truxene 1, 372 nm light was detected under excitation at 351 nm, with a fluorescence quantum yield of 18.4% (Fig. 6a). Notably, bright orange emission at 580 nm (*λ*_ex_ = 360 nm, Fig. 6b) is observed in the monoanionic state (Na-1^−^), accompanied by higher emission intensity compared to 1 (*Φ* = 36.4%) with a decay time of 17.6 ns. In the two-fold deprotonated product Na_2_-1^2−^, a bathochromic shift with an orange–red emission appears at 608 nm (*λ*_ex_ = 351 nm, Fig. 6c) with similar emission intensity but a longer fluorescence lifetime (31.1 ns). In contrast, the fluorescence maximum of 6 is shifted back to 550 nm compared toNa_2_-1^2−^, accompanied by a decrease in quantum yield to *Φ* = 1.7% (Fig. 6d). The large Stokes shift values in Na-1^−^, Na_2_-1^2−^, and K_3_-1^3−^ are attributed to increasing π-conjugation, which increases the absorption coefficient and allows a higher chance of excitation for π-electrons.^16^ The decay constants *k*_f_ and *k*_nr_ were calculated and compared for 1, Na-1^−^, Na_2_-1^2−^, and K_3_-1^3^. As shown in Table 2, Na-1^−^ is expected to exhibit a higher *Φ*_f_ value among the charged species due to its faster spontaneous emission rate compared to Na_2_-1^2−^ and K_3_-1^3−^. Compound 1 exhibits a substantially higher nonradiative decay rate (*k*_nr_), affording a relatively lower quantum yield. Conversely, the low quantum yield observed in K_3_-1^3−^ (1.7%) can be attributed to a diminished emission rate (0.003 ns^−1^) and a more rapid nonradiative decay rate (0.176 ns^−1^).

**Table 2:** Absorption and emission properties of 1, Na-1^−^, Na_2_-1^2−^, and K_3_-1^3−^

Compound	*λ*_abs_/nm	*λ*_em_/nm	*Φ*_f_	〈*τ*_f_〉/ns	*k*_f_/ns^−1^	*k*_nr_/ns^−1^
1	300	372	0.184	2.9	0.063	0.281
Na-1^−^	332	580	0.364	17.6	0.021	0.036
Na_2_-1^2−^	338	608	0.323	31.1	0.010	0.022
K_3_-1^3−^	370	550	0.017	5.6	0.003	0.176

## Conclusions

Using different alkali metals, we demonstrated that truxene can lose up to three protons through a controlled stepwise deprotonation process. Products of truxenyl anions with varying negative charges, namely monoanion, dianion, and trianion, have been isolated with different counterions and crystallographically characterized. A clear variation in alkali metal ion binding patterns was observed for light (Na^+^) *vs.* heavy (K^+^ and Cs^+^) counterions. The structural analysis revealed a notable core curvature of truxenyl anions affected by negative charge and metal coordination. The ^1^H NMR results indicated a change in aromaticity during stepwise deprotonation, as illustrated by notable upfield shifts of protons on the three five-membered rings. Moreover, computational NICS and ACID analyses were performed for truxenyl anions with different charging states with direct relevance to the experimental studies.

We also observed that the increase in charge and aromaticity causes a change in optical properties, which was supported by UV-vis and photoluminescence spectroscopic investigations. The increase in aromaticity and π-conjugation yielded a red shift in the absorption and photoluminescent spectra. In particular, large Stokes shifts were observed in the truxenyl monoanion and dianion with a higher emission quantum yield and time of decay. Overall, stepwise deprotonation of truxene provides the first crystallographically characterized examples of truxenyl anions with different charges and charge-dependent optical properties. The successful generation of truxenyl anions with three different negative charges opens up their broad use in ligand exchange reactions and enables expansion of their organometallic and coordination chemistry.

## Data availability

Data available in the ESI: Synthetic procedures, NMR, UV-vis, PL and TA spectra, X-ray crystallographic data and computational details.†

## Author contributions

Y. G. and H. S. T. synthesized the anionic products, completed their detailed structural descriptions and product characterizations; Y. Z. provided computational data analysis and wrote the corresponding manuscript sections; Z. W. and Z. Z. performed the X-ray data collection and refinement; H. H. contributed to the discussion and presentation of results; M. A. P. and Z. Z. conceived and supervised this work. Z. Z. carried out the theoretical calculations and drafted the manuscript with the support and contribution from all authors.

## Conflicts of interest

There are no conflicts to declare.

## Supplementary Material

SC-014-D3SC04885C-s001

SC-014-D3SC04885C-s002
