# Supplementary material for: Stepwise deprotonation of truxene: structures, metal complexation, and charge-dependent optical properties
Source: Chem Sci. 2023 Oct 31;14(45):13219–27. doi: 10.1039/d3sc04885c (PMC10664571; doi:10.1039/d3sc04885c)
Supplement: SC-014-D3SC04885C-s001 [file SC-014-D3SC04885C-s001.pdf]

Electronic Supplementary Material (ESI) for Chemical Science.
This journal is © The Royal Society of Chemistry 2023

Supporting information

Stepwise deprotonation of truxene: Structures, metal complexation and charge-dependent optical properties

Yumeng Guo,[a] Herdya S Torchon,[b] Yikun Zhu,[b] Zheng Wei,[b] Zhenyi Zhang,[c] Haixiang Han,[a,*] Marina A. Petrukhina[b,*], Zheng Zhou[a,*]

[a] *School of Materials Science and Engineering, Tongji University, Shanghai 201804, China*
[b] *Department of Chemistry, University at Albany, State University of New York, Albany, NY 12222, USA*
[c] *Bruker (Beijing) Scientific Technology Co.,Ltd. Shanghai 200233, China*

Table of Contents

I. **Methods and Materials** S2

II. **NMR Spectroscopic Investigation** S6

III. **UV-Vis Absorption Spectroscopic Investigation** S11

IV. **Photoluminance Spectroscopic Investigation** S16

V. **Transient Absorption Spectroscopic Investigation** S18

VII. **Crystal Structure Solution and Refinement Details** S20

VIII. **Computational Details** S32

IX. **References** S46

I. Methods and Materials

All manipulations were carried out using break-and-seal and glove-box techniques under an atmosphere of argon.[1] Tetrahydrofuran (THF, 99%, Sigma Aldrich) and hexanes (99%, Sigma Aldrich) were dried over NaK_2 alloy and distilled prior to use. THF-d_8 (99%, Sigma Aldrich) was dried over NaK_2 alloy and vacuum-transferred. Sodium (98%, Sinopharm), potassium (98%, Sinopharm), cesium (99.5%, Sigma Aldrich), 18-crown-6 ether (99%, Sigma Aldrich), and *n*-BuLi (2.5M solution in hexanes, Adamas) were used as received. Truxene ($C_{27}H_{18}$, 98%, Adamas) was purified by sublimation at 265 °C prior to use.

^{1}H NMR spectra of **1, Na-1**$^-$, **Na**$_2$**-1**$^{2-}$, and **K**$_3$**-1**$^{3-}$ were measured using Bruker Avance 400 spectrometer, and ^{1}H NMR spectra of **Cs-1**$^-$ and **Cs**$_2$**-1**$^{2-}$ were measured using a Bruker Ascend 500 spectrometer. All ^{1}H NMR spectra were referenced to the resonances of the corresponding solvent used. UV-vis absorption spectra of **1, Na-1**$^-$, **Na**$_2$**-1**$^{2-}$, and **K**$_3$**-1**$^{3-}$ were recorded on an Agilent Cary-3500 UV-Vis-NIR spectrometer, and UV-vis absorption spectra of **Cs-1**$^-$ and **Cs**$_2$**-1**$^{2-}$ were recorded on a Shimadzu UV-2600i UV-vis spectrophotometer. The solution sample for UV-vis was sealed in a glass ampule (O.D. 1.2 cm) under argon. Photoluminescence (PL) spectra were measured on an Edinburgh FLS980 spectrometer. The solution sample for PL was sealed in a glass ampule (O.D. 1.2 cm, L. 4.5 cm) under argon and covered with aluminum foil prior to use. Femtosecond transient absorption spectra (fs-TA) were measured on an Ultrafast System Helios spectrometer. A small portion of the laser fundamental was focused into a sapphire plate to produce a supercontinuum in the visible range, which overlapped with the pump in time and space. Multiwavelength transient spectra were recorded at different pump-probe delay times (Helios Fire, Ultrafast Systems). Time zero, solvent response, and chirp corrections were employed using software supplied by Ultrafast Systems. The solution sample for fs-TA was sealed in a 1-mm glass cuvette with an air-free Teflon plug under argon and covered with aluminum foil prior to use. Element analysis of truxenyl anions was not performed due to their high air/moisture sensitivity.

Crystallization of $C_{27}H_{18}$ (1)

10 mg of truxene (0.030 mmol) was sealed in a glass tube (8 cm) under vacuum. The tube was placed in a furnace with the temperature set to 265 °C for sublimation. After 5 days, yellow needle-shaped single crystals suitable for single crystal X-ray diffraction were formed. Yield: 6.7

mg, 67%. ^{1}H NMR (THF-d_8, δ, ppm, 25 °C): 4.32 (6H, $C_{27}H_{18}$), 7.35 (3H, $C_{27}H_{18}$), 7.46 (3H, $C_{27}H_{18}$), 7.69 (3H, $C_{27}H_{18}$), 8.01 (3H, $C_{27}H_{18}$). UV-Vis (THF, λ_{max}, nm): 300, 325, 334.

Preparation of [Na$^+$(18-crown-6)(THF)$_2$][$C_{27}H_{17}^-$]·2THF (Na-1$^-$)

THF (2.0 mL) was added to a customized glass system containing excess Na metal (10.0 mg, 0.435 mmol), truxene (10.4 mg, 0.0304 mmol, sublimed) and 18-crown-6 (8.1 mg, 0.0308 mmol). The reaction was stirred for 12 hours at 25 °C under argon. The initial pale-yellow color (neutral ligand) changed to orange after 2 hours (monooanion) and remained the same color before the reaction was stopped. The mixture was then filtered, and the orange filtrate was layered with 1.0 mL of hexanes. The ampule was sealed under argon and stored at –20 °C. Orange plates were present after 7 days. Yield: 24 mg, 85%. ^{1}H NMR (THF-d_8, δ, ppm, 25 °C): 4.14 (2H, $C_{27}H_{17}^-$), 4.29 (2H, $C_{27}H_{17}^-$), 6.62 (1H, $C_{27}H_{17}^-$), 6.64 (1H, $C_{27}H_{17}^-$), 6.88 (2H, $C_{27}H_{17}^-$), 6.99 (1H, $C_{27}H_{17}^-$), 7.19 (1H, $C_{27}H_{17}^-$), 7.31 (1H, $C_{27}H_{17}^-$), 7.42 (1H, $C_{27}H_{17}^-$), 7.48 (2H, $C_{27}H_{17}^-$), 7.62 (1H, $C_{27}H_{17}^-$), 8.01 (1H, $C_{27}H_{17}^-$), 8.05 (1H, $C_{27}H_{17}^-$). UV-Vis (THF, λ_{max}, nm): 332, 441, 548.

Preparation of [{Na$^+$(18-crown-6)(THF)$_2$}$_2$($C_{27}H_{16}^{2-}$)] (Na$_2$-1^{2-})

THF (2.0 mL) was added to a customized glass system containing excess Na metal (10.0 mg, 0.435 mmol), truxene (10.4 mg, 0.0304 mmol, sublimed) and 18-crown-6 (16.3 mg, 0.0617 mmol). After ultrasonication for 15 seconds, the reaction was stirred for 3 days at 25 °C under argon. The initial pale-yellow color (neutral ligand) changed to orange in 30 minutes (monoanion), and finally changed to red brown (dianion). The mixture was then filtered, and the red brown filtrate was layered with 1.0 mL of hexanes. The ampule was sealed under argon and stored at – 20 °C. Red plates were present after 7 days. Yield: 29 mg, 80%. ^{1}H NMR (THF-d_8, δ, ppm, 25 °C): 4.24 (2H, $C_{27}H_{16}^{2-}$), 6.57 (1H, $C_{27}H_{16}^{2-}$), 6.64 (1H, $C_{27}H_{16}^{2-}$), 6.75 (4H, $C_{27}H_{16}^{2-}$), 6.78 (1H, $C_{27}H_{16}^{2-}$), 7.21 (1H, $C_{27}H_{16}^{2-}$), 7.36 (1H, $C_{27}H_{16}^{2-}$), 7.41 (1H, $C_{27}H_{16}^{2-}$), 7.54 (1H, $C_{27}H_{16}^{2-}$) , 7.90 (2H, $C_{27}H_{16}^{2-}$), 8.33 (1H, $C_{27}H_{16}^{2-}$). UV-Vis (THF, λ_{max}, nm): 338, 452, 511, 549.

Preparation of [{Cs$^+$(18-crown-6)}($C_{27}H_{17}^-$)] (Cs-1$^-$)

THF (1.5 mL) was added to a customized glass system containing Cs metal (1.2 mg, 0.009 mmol), truxene (3.0 mg, 0.009 mmol, sublimed) and 18-crown-6 (5.0 mg, 0.018 mmol). The reaction mixture was stirred at 25 °C under argon for 30 minutes. The initial pale-yellow color

(neutral ligand) changed to orange after 5 minutes and remained the same until the reaction was stopped. The mixture was then filtered, and the orange filtrate was layered with 1.0 mL of anhydrous hexanes. A bright-orange color and no precipitate were observed on the filter after filtration. The ampule was sealed under argon and stored at 5 °C. After 14 days, dark-brown block-shaped crystals were observed in the ampule. Yield: 4 mg, 60%. ^{1}H NMR (THF-d_8, δ, ppm, 25 °C): 4.23 (2H, $C_{27}H_{17}^{-}$), 4.37 (2H, $C_{27}H_{17}^{-}$), 6.77 (1H, $C_{27}H_{17}^{-}$), 6.81 (1H, $C_{27}H_{17}^{-}$), 6.97 (1H, $C_{27}H_{17}^{-}$), 7.02 (1H, $C_{27}H_{17}^{-}$), 7.09 (1H, $C_{27}H_{17}^{-}$), 7.27 (1H, $C_{27}H_{17}^{-}$), 7.40 (1H, $C_{27}H_{17}^{-}$), 7.50 (1H, $C_{27}H_{17}^{-}$), 7.55 (1H, $C_{27}H_{17}^{-}$), 7.62 (1H, $C_{27}H_{17}^{-}$), 7.72 (1H, $C_{27}H_{17}^{-}$), 8.15 (2H, $C_{27}H_{17}^{-}$). UV-Vis (THF, λ_{max}, nm): 332, 388 (sh), 436, 529.

Preparation of [{Cs^+(18-crown-6)}$_2$($C_{27}H_{16}^{2-}$)] (Cs_2-1^{2-})

THF (1.5 mL) was added to a customized glass system containing Cs metal (4.0 mg, 0.030 mmol) and truxene (5.0 mg, 0.015 mmol, sublimed). The reaction was stirred at 25 °C under argon for 1 hour. The initial pale-yellow color (neutral ligand) changed to yellow in 15 minutes and deepened to orange after 30 minutes. The mixture was then filtered, and the orange filtrate was layered with 1.5 mL of hexanes containing 18-crown-6 (10.0 mg, 0.038 mmol). The ampule was sealed under argon and stored at 5 °C. Orange plates were present after 5 days. Yield: 12.0 mg, 70%. ^{1}H NMR (THF-d_8, δ, ppm, 25 °C): 4.22 (2H, $C_{27}H_{16}^{2-}$), 6.56 (1H, $C_{27}H_{16}^{2-}$), 6.67 (1H, $C_{27}H_{16}^{2-}$), 6.74 (5H, $C_{27}H_{16}^{2-}$), 7.21 (1H, $C_{27}H_{16}^{2-}$), 7.35 (1H, $C_{27}H_{16}^{2-}$), 7.41 (1H, $C_{27}H_{16}^{2-}$), 7.56 (1H, $C_{27}H_{16}^{2-}$) , 7.86 (2H, $C_{27}H_{16}^{2-}$), 8.23 (1H, $C_{27}H_{16}^{2-}$). UV-Vis (THF, λ_{max}, nm): 332, 386, 446, 498, 533.

Preparation of [K^+(18-crown-6)(THF)$_2$][{K^+(18-crown-6)}$_2$($C_{27}H_{15}^{3-}$)]·THF (K_3-1^{3-})

THF (2.0 mL) was added to a customized glass system containing excess K metal (10.0 mg, 0.43 mmol), truxene (3.0 mg, 0.009 mmol, sublimed) and 18-crown-6 (9.6 mg, 0.036 mmol). The reaction mixture was stirred at 25 °C under argon for 2 hours. The initial pale-yellow color (neutral ligand) immediately changed to orange (monoanion), orange brown (dianion) after 10 minutes, and finally deepened to dark green after 20 minutes and remained the same color until the reaction was stopped. The mixture was then filtered, and the dark green filtrate was layered with 1.0 mL of anhydrous hexanes. The ampule was sealed under argon and stored at 5 °C. After 3 days, yellow block-shaped crystals were observed in the ampule. Yield: 10 mg, 80%. ^{1}H NMR

(THF-d_8, δ, ppm, 25 °C): 6.58 (6H, $C_{27}H_{15}^{3-}$), 6.76 (3H, $C_{27}H_{15}^{3-}$), 7.43 (3H, $C_{27}H_{15}^{3-}$), 8.14 (3H, $C_{27}H_{15}^{3-}$). UV-Vis (THF, λ_{max}, nm): 370, 426, 498.

II. NMR Spectroscopic Investigation

Sample preparation: Crystals of **1** (~2.0 mg) were dissolved in THF-d_8 (~0.6 mL). Crystals of **Na-1**$^{-}$, **Na$_2$-1**$^{2-}$, **Cs-1**$^{-}$, **Cs$_2$-1**$^{2-}$**,** and **K$_3$-1**$^{3-}$ (~5.0 mg) were dried *in-vacuo* and dissolved in THF-d_8 (~0.6 mL). The resulting solutions were transferred to NMR tubes which were sealed under argon. The *in situ* generated $C_{27}H_{15}^{3-}$ anions were prepared by mixing 3.0 mg of **1** with 0.1 ml of *n*-BuLi/excess Cs metal, which were then sealed under argon. After standing for 5 days, the samples were tested for ^{1}H NMR spectrum of *in situ* generated $C_{27}H_{15}^{3-}$. The ^{1}H NMR spectra were collected shown as Figs. S1−S8.

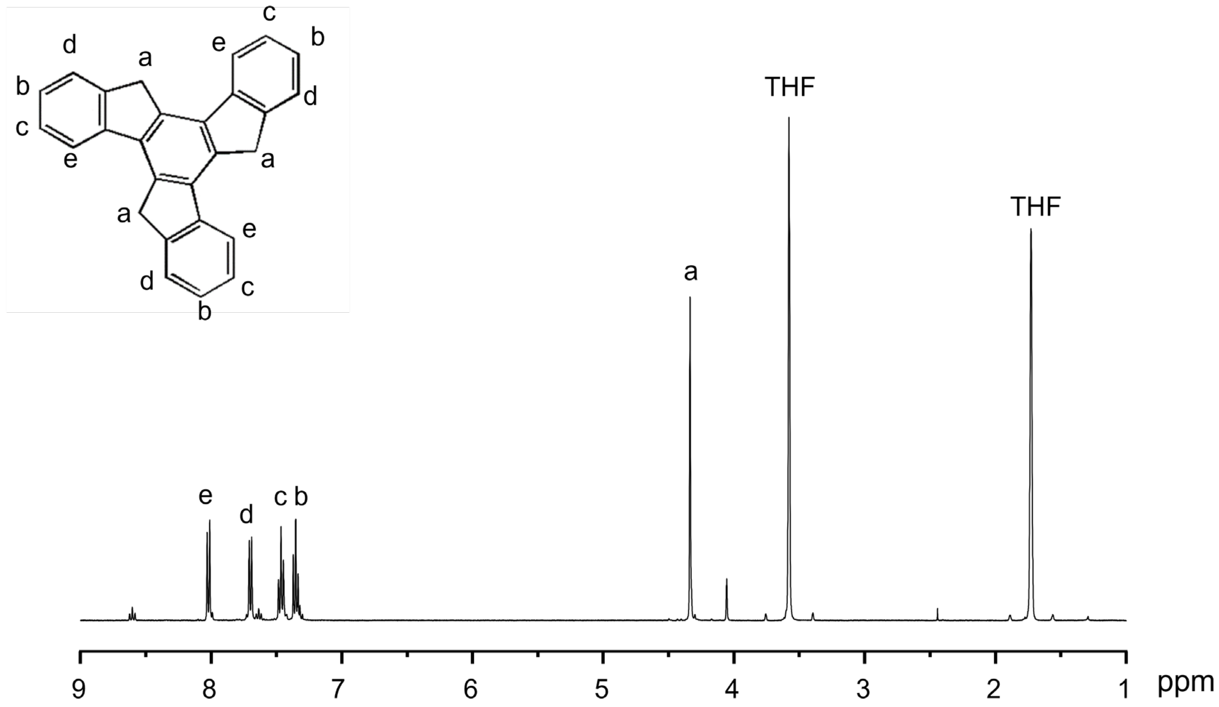

Fig. S1 ^{1}H NMR spectrum of **1** in THF-d_8 at 25 °C.

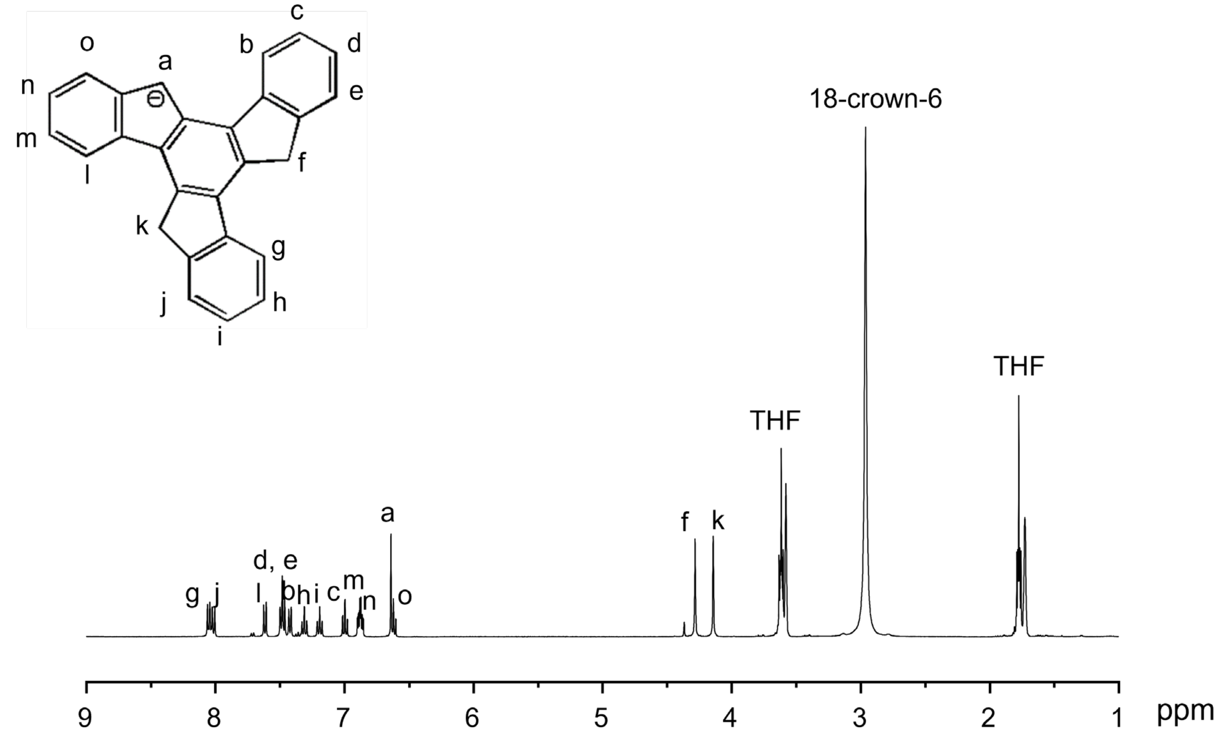

Fig. S2 ^{1}H NMR spectrum of **Na-1**$^{-}$ in THF-d_8 at 25 °C.

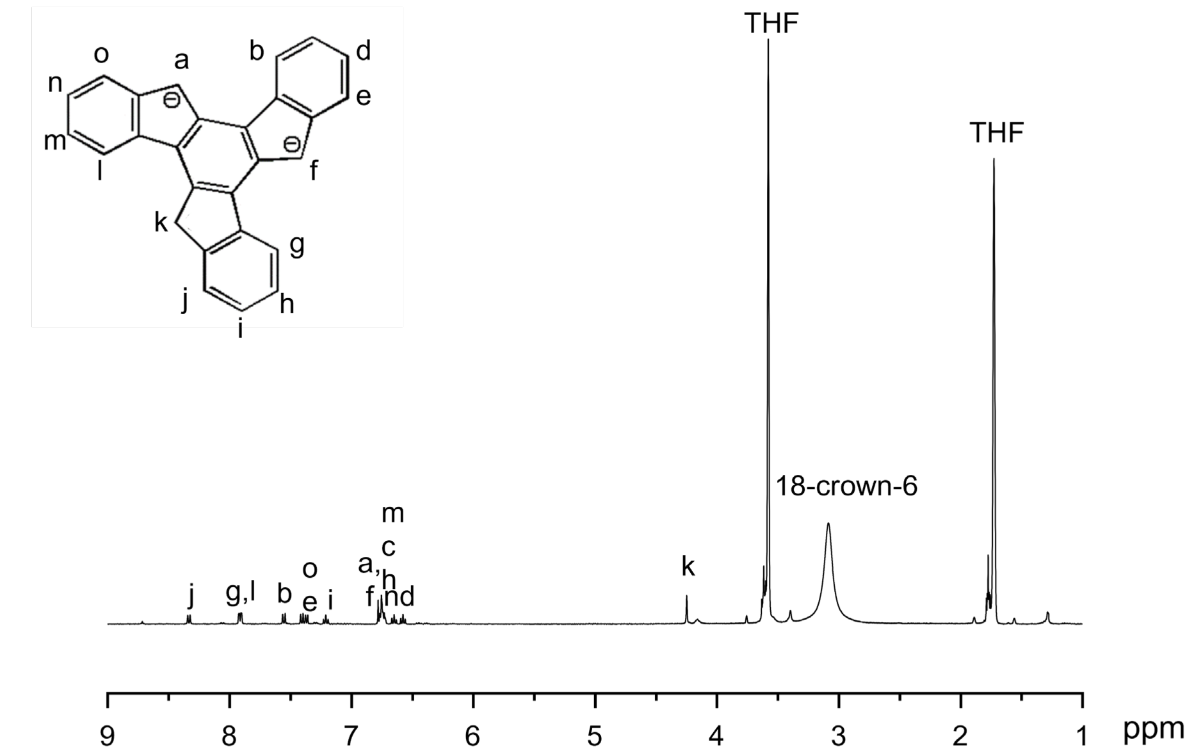

Fig. S3 ^{1}H NMR spectrum of **Na$_2$-1^{2-}** in THF-d_8 at 25 °C.

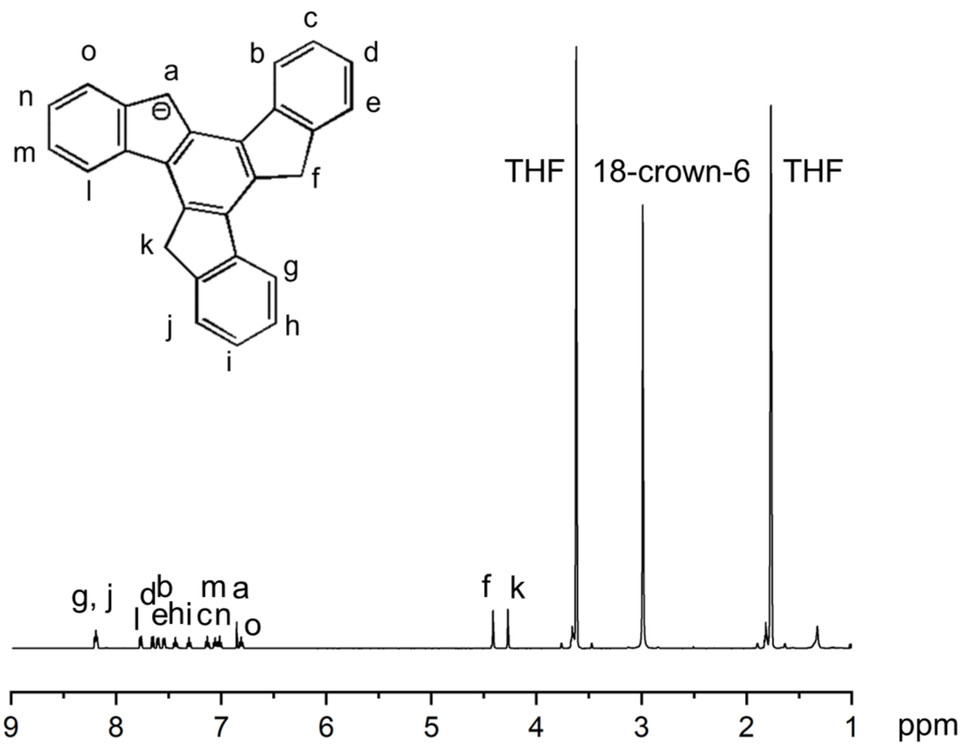

Fig. S4 ^{1}H NMR spectrum of **Cs-1^{-}** in THF-d_8 at 25 °C.

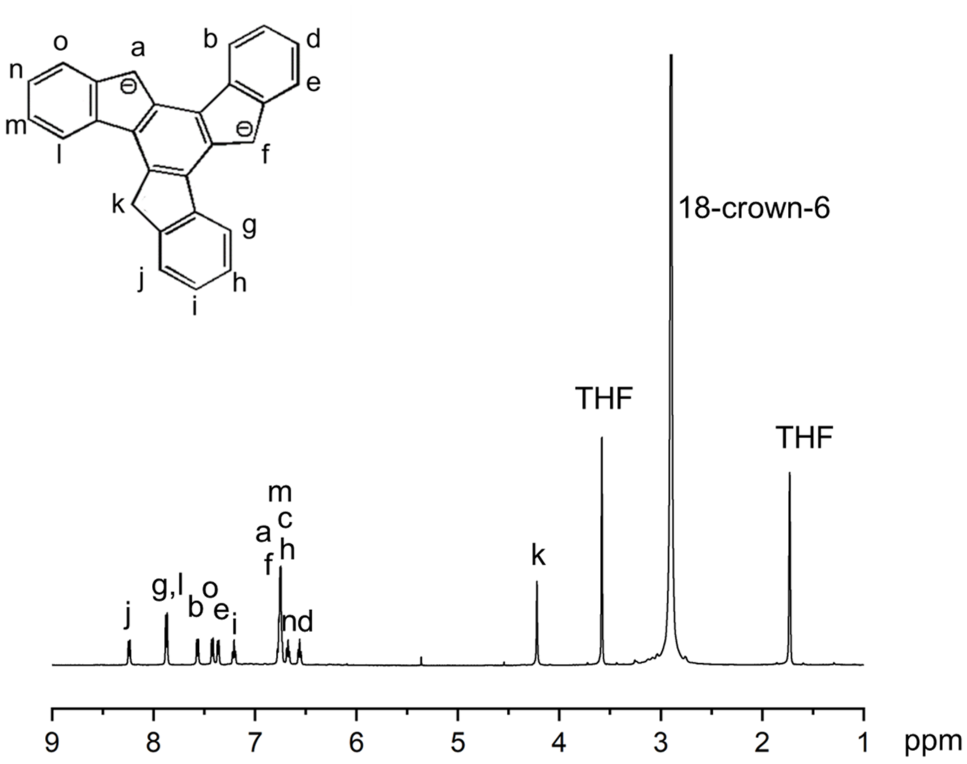

Fig. S5 ^{1}H NMR spectrum of **Cs$_2$-1^{2-}** in THF-d_8 at 25 °C.

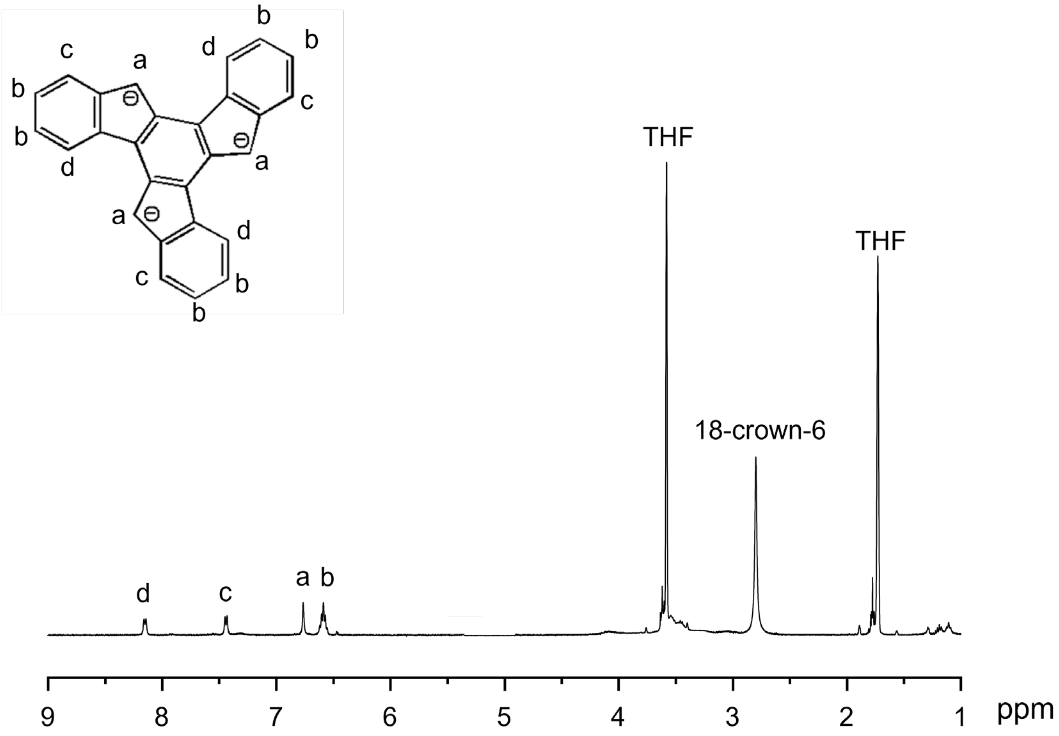

Fig. S6 ^{1}H NMR spectrum of **K_3-1^{3-}** in THF-d_8 at 25 °C.

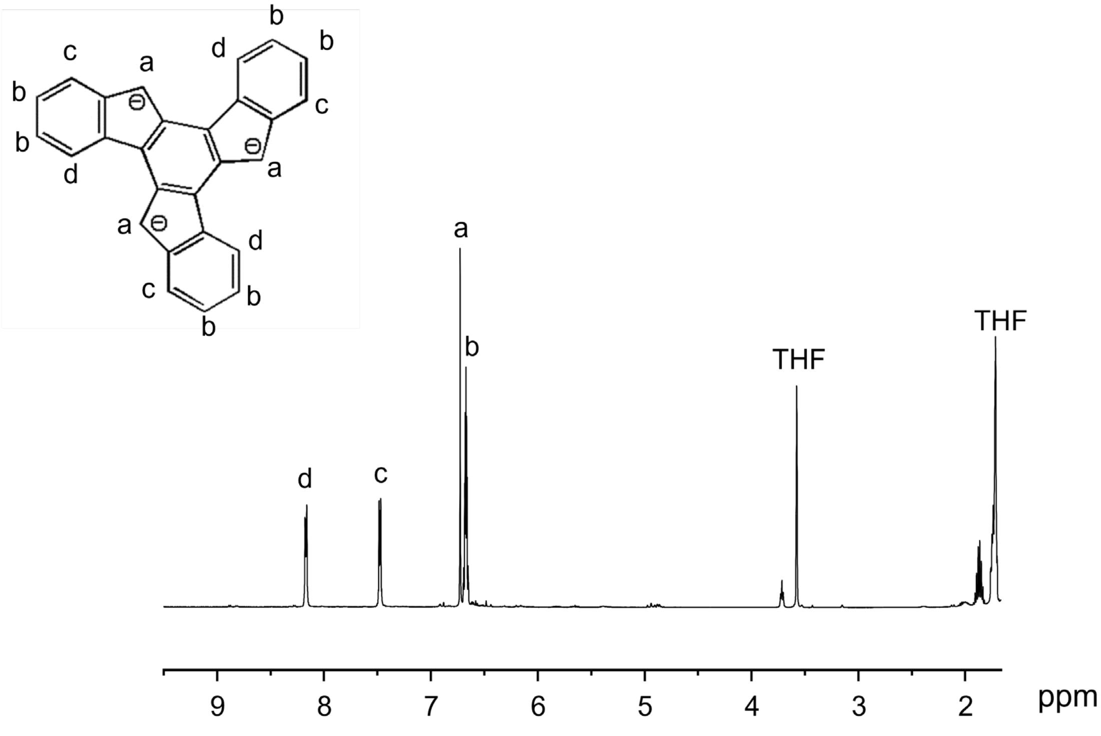

Fig. S7 ^{1}H NMR spectrum of *in situ* generated $C_{27}H_{15}^{3-}$ by *n*-BuLi in THF-d_8 at 25 °C.

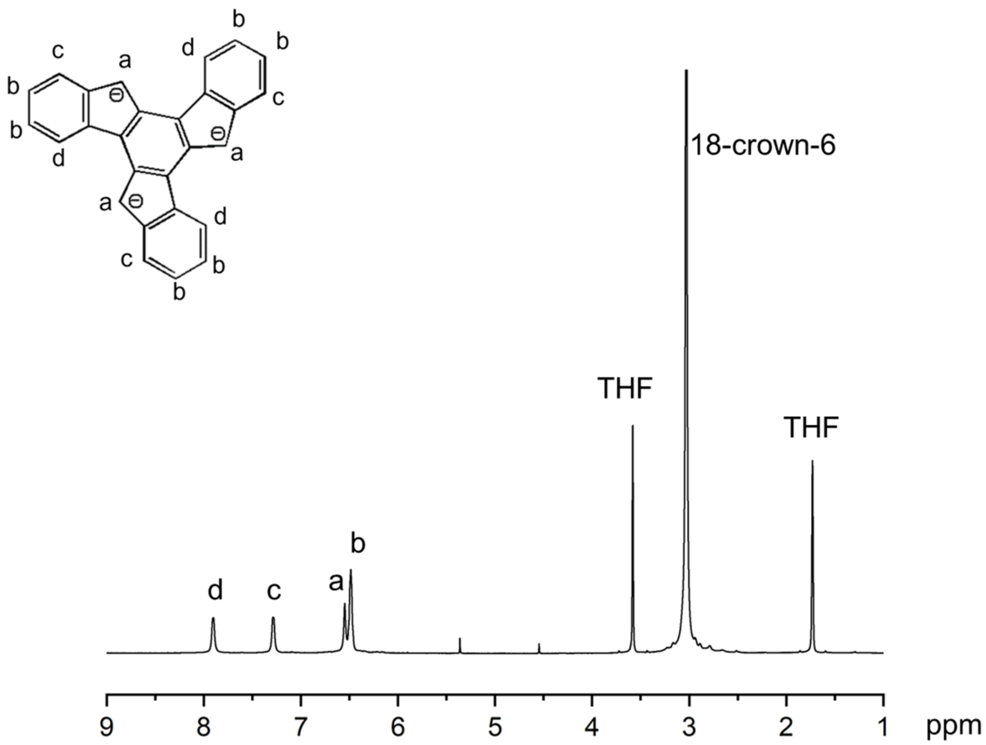

Fig. S8 ^{1}H NMR spectrum of *in situ* generated $C_{27}H_{15}^{3-}$ by Cs in THF-d_8 at 25 °C.

III. UV-Vis Absorption Spectroscopic Investigation

Sample preparation: Crystals of **1**, **Na-1**$^-$, **Na$_2$-1**$^{2-}$, **Cs-1**$^-$, **Cs$_2$-1**$^{2-}$, and **K$_3$-1**$^{3-}$ (~0.20 mg) were dried *in-vacuo*, dissolved in 1.0 mL of anhydrous THF, and transferred into a 1 mm glass cuvette. The ampule was sealed under argon, and UV-vis spectra were collected at room temperature.

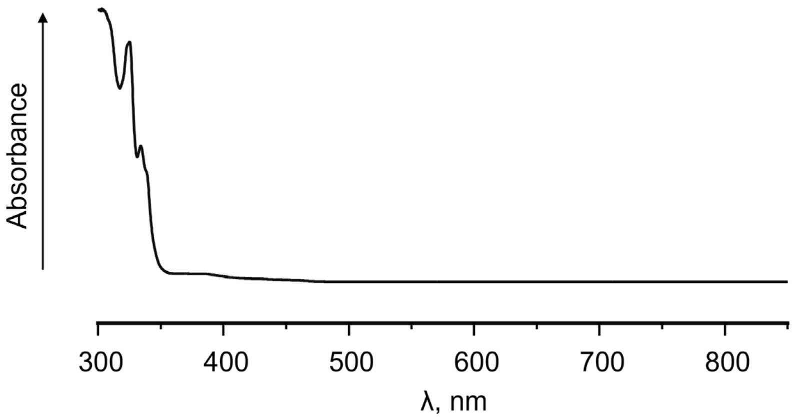

Fig. S9 UV-vis spectrum of **1** in THF at 25 °C.

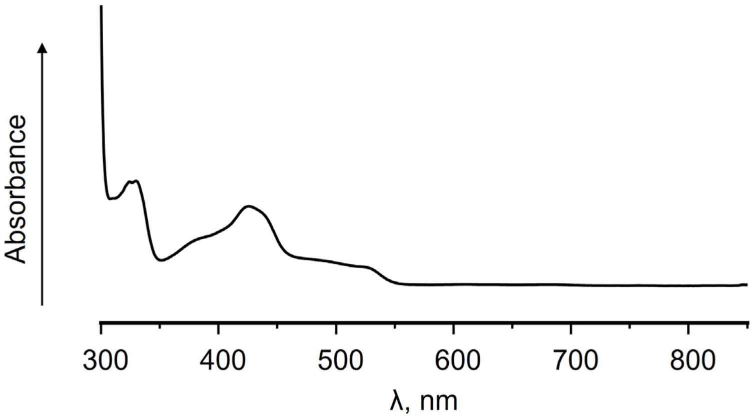

Fig. S10 UV-vis spectrum of **Na-1**$^-$ in THF at 25 °C.

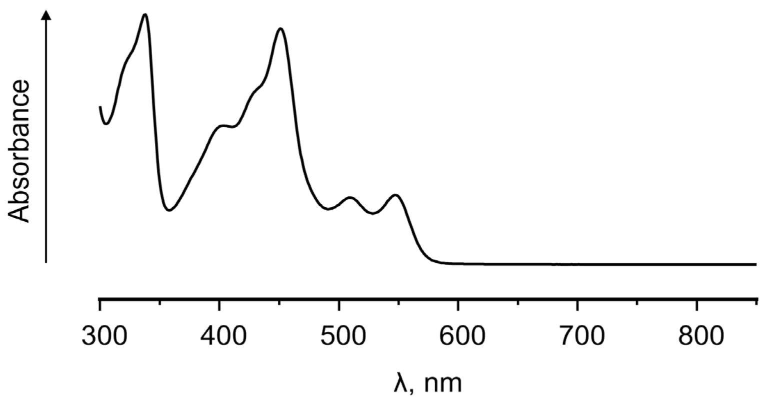

Fig. S11 UV-vis spectrum of $\mathbf{Na_2\text{-}1^{2-}}$ in THF at 25 °C.

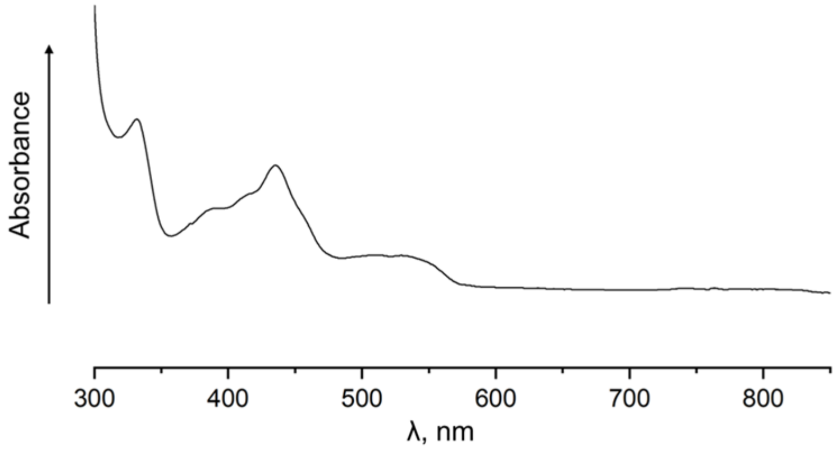

Fig. S12 UV-vis spectrum of $\mathbf{Cs\text{-}1^{-}}$ in THF at 25 °C.

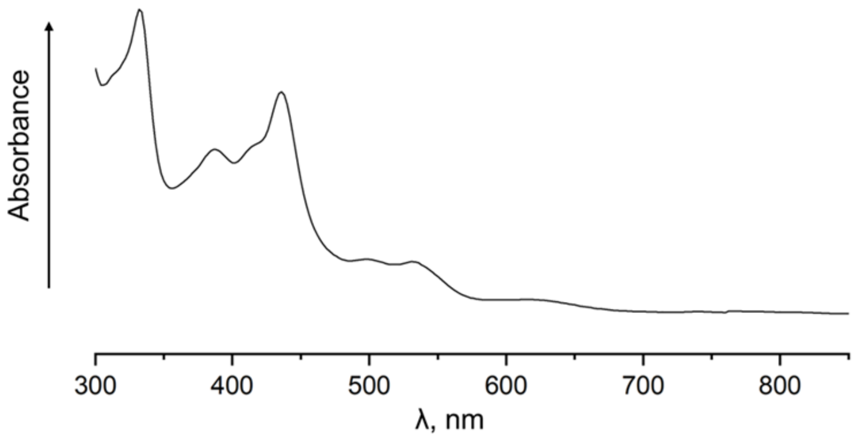

Fig. S13 UV-vis spectrum of $\mathbf{Cs_2\text{-}1^{2-}}$ in THF at 25 °C.

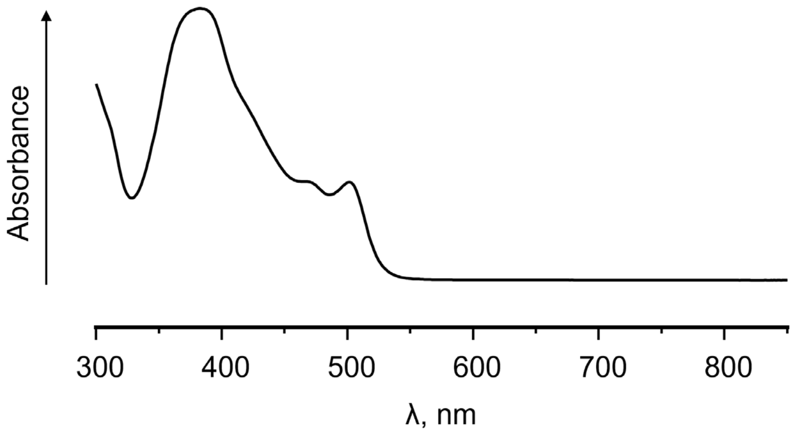

Fig. S14 UV-vis spectrum of **K₃-1**$^{3-}$ in THF at 25 °C.

According to the Lambert-Beer Law, light absorption is directly proportional to path length and the concentration of the solution, which is written as:

$$\boldsymbol{A} = \boldsymbol{\alpha} \cdot \boldsymbol{l} \cdot \boldsymbol{c}$$

where $\boldsymbol{A}$ stands for absorbance, $\boldsymbol{\alpha}$ stands for absorptivity, $\boldsymbol{l}$ stands for path length, and $\boldsymbol{c}$ stands for concentration. When cm is used for $\boldsymbol{l}$ and M is used for $\boldsymbol{c}$, $\boldsymbol{\alpha}$ can be replaced by $\boldsymbol{\varepsilon}$, which is also called molar absorptivity.

In order to acquire a more accurate value of $\boldsymbol{\varepsilon}$, a five-point $\boldsymbol{A}$–$\boldsymbol{c}$ data correction was employed. First, the concentration-dependent UV-vis spectra were collected at room temperature. Second, a linear fitting was generated using five sets of absorbance and concentration values. Third, pick up a concentration and use its corrected absorbance valve for calculation.

No.	**1** ($C_{27}H_{18}$)	**Na-1**$^{-}$ ($C_{27}H_{17}^{-}$)	**Na**$_2$**-1**$^{2-}$ ($C_{27}H_{16}^{2-}$)	**K**$_3$**-1**$^{3-}$ ($C_{27}H_{15}^{3-}$)
$\boldsymbol{\varepsilon}$	12402	34579	32873	20531

Note: Values were calculated using largest concentration with A < 1. Because the decomposition of **2** always occurs during dilution, this correction was not performed for **2**.

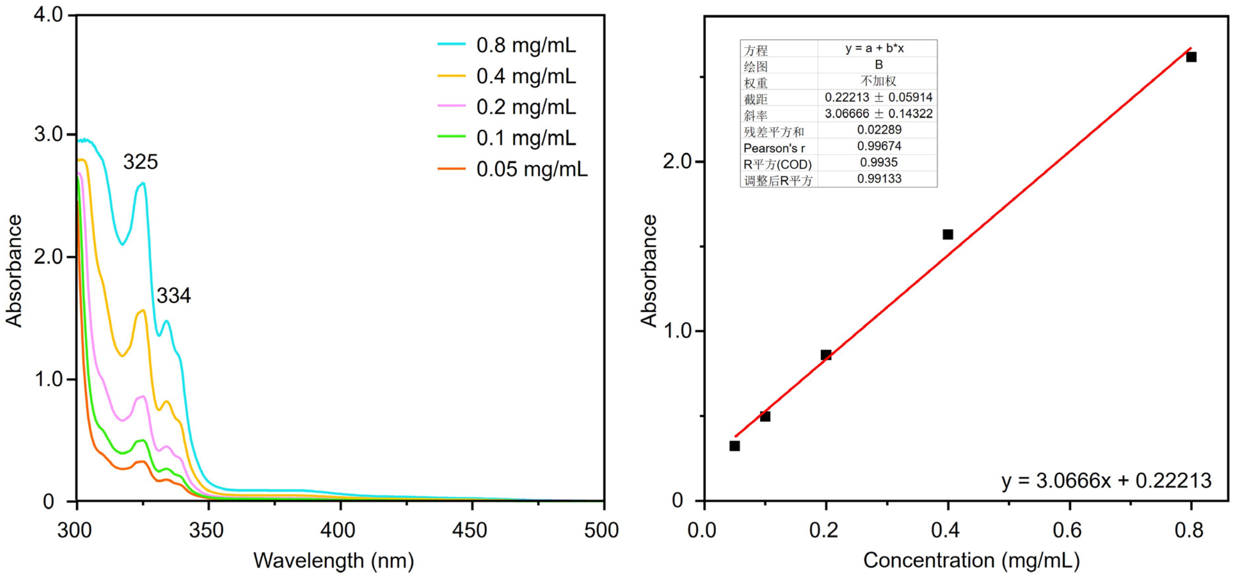

Fig. S15 Concentration-dependent UV-vis spectra of **1** in THF at 25 °C and its absorption coefficient correction.

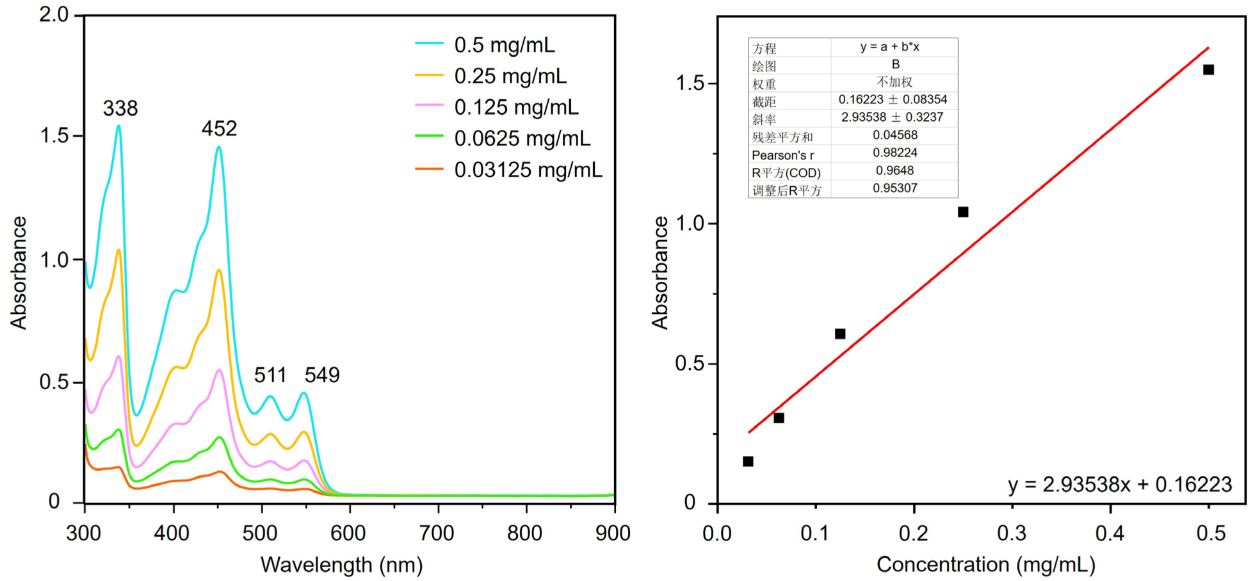

Fig. S16 Concentration-dependent UV-vis spectra of **Na_2-1^{2-}** in THF at 25 °C and its absorption coefficient correction.

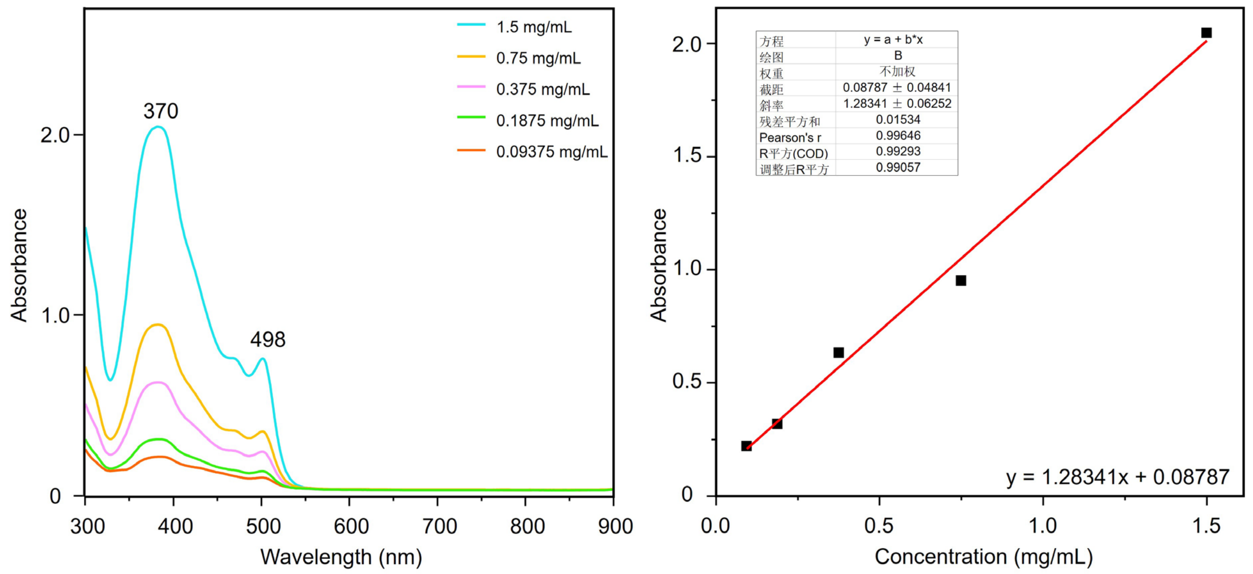

Fig. S17 Concentration-dependent UV-vis spectra of **K_3-1^{3-}** in THF at 25 °C and its absorption coefficient correction.

IV. Photoluminance Spectroscopic Investigation

Sample preparation: Crystals of **1** (~5.0 mg) were dissolved in THF (2.0 mL), crystals of **Na-1**$^{-}$, **Na**$_2$**-1**$^{2-}$, and **K**$_3$**-1**$^{3-}$ (~5.0 mg) were dried *in-vacuo* and dissolved in anhydrous THF (2.0 mL). The resulting solution was sealed under argon in a glass ampule (O.D. 1.0 cm, L. 4.5 cm), and the excitation and emission spectra were collected at room temperature.

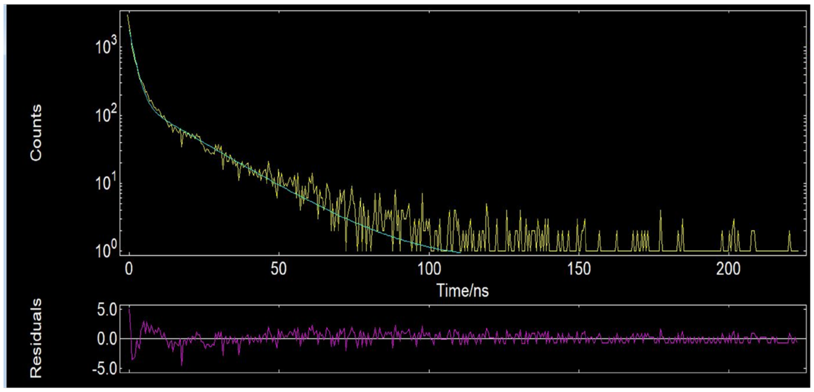

λ = 375 nm	
τ_1	1.7690 ns
τ_2	16.7987 ns
B_1	2131.4736
B_2	175.0702
R_1	0.9241
R_2	0.0759
$\langle\tau\rangle$ = 2.9098 ns	

$R_i = B_i / (B_1+B_2)$

$\langle\tau\rangle = R_1 \cdot \tau_1 + R_2 \cdot \tau_2$

Fig. S18 Bi-exponential fitting of PL decay curve of **1** recorded at λ_{em} = 375 nm and the calculated parameters.

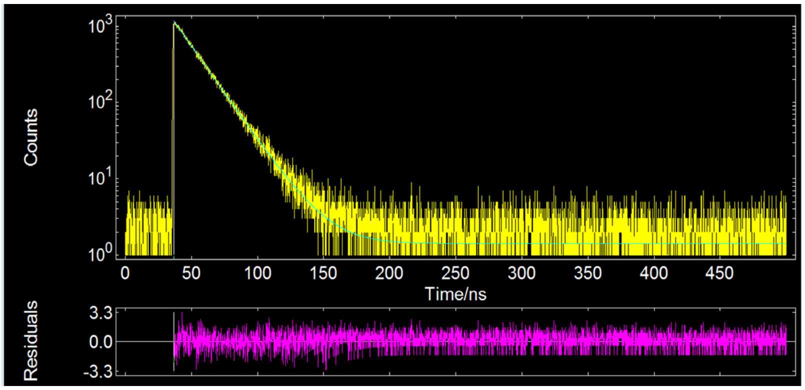

λ = 576 nm	
τ_1	17.58 ns
B_1	1.175
R_1	1.175
$\langle\tau\rangle$ = 17.6 ns	

$R_i = B_i / (B_1+B_2)$

$\langle\tau\rangle = R_1 \cdot \tau_1 + R_2 \cdot \tau_2$

Fig. S19 Bi-exponential fitting of PL decay curve of **Na-1**$^{-}$ recorded at λ_{em} = 576 nm and the calculated parameters.

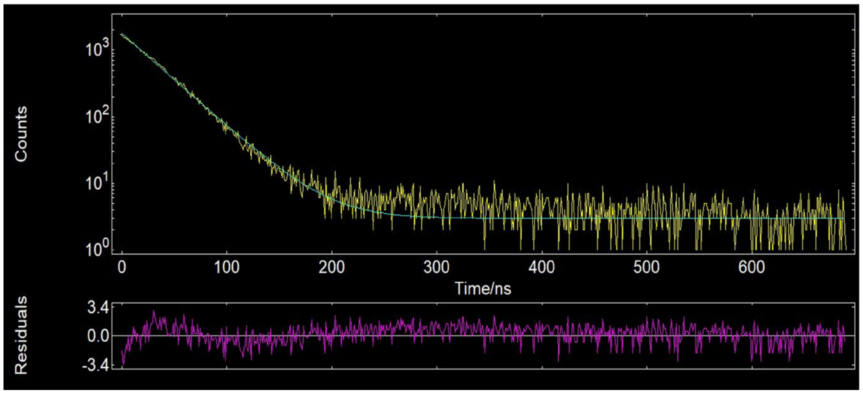

λ = 608 nm	
τ_1	31.0781 ns
B_1	1817.7809
R_1	1817.7809
$\langle\tau\rangle$ = 31.0781 ns	

$R_i = B_i / (B_1+B_2)$

$\langle\tau\rangle = R_1 \cdot \tau_1 + R_2 \cdot \tau_2$

Fig. S20 Bi-exponential fitting of PL decay curve of **Na_2-1^{2-}** recorded at λ_{em} = 608 nm and the calculated parameters.

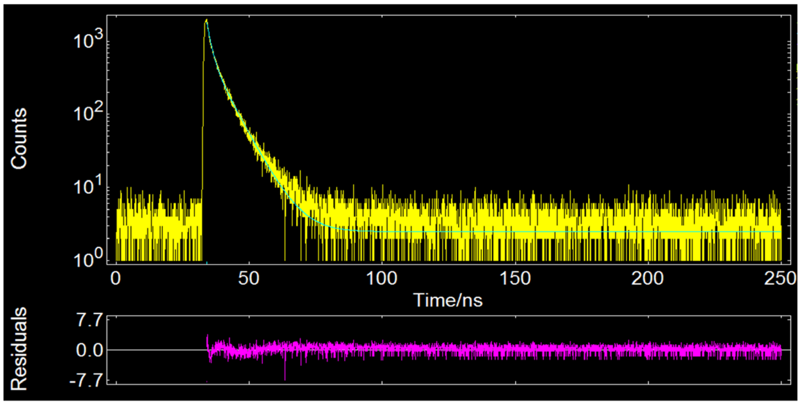

λ = 550 nm	
τ_1	1.543 ns
τ_2	6.325 ns
B_1	1.182
B_2	6.588
R_1	0.1521
R_2	0.8479
$\langle\tau\rangle$ = 5.5977 ns	

$R_i = B_i / (B_1+B_2)$

$\langle\tau\rangle = R_1 \cdot \tau_1 + R_2 \cdot \tau_2$

Fig. S21 Bi-exponential fitting of PL decay curve of **K_3-1^{3-}** recorded at λ_{em} = 550 nm and the calculated parameters.

V. Transient Absorption Spectroscopic Investigation

Femtosecond transient absorption spectroscopy (fs-TA) is a technique that can provide more details for excited species. Equipped with a white light probe source and a single-wavelength pump laser, the spectrometer detects the absorption difference (ΔOD) between the pump laser signal and probe signal. By adjusting the time delay of the pump laser pulse, one provides the time-resolved absorption spectrum at each time gap. There are mainly three components: ground-state bleach (GSB, a negative signal where the pump pulse depletes the ground state, excited-state absorption (ESA, a positive signal caused by energy absorption under excitation), and stimulated emission (SE, a negative signal close to the emission range). As shown in Fig. S22 bottom, the top curves describe the transient absorption states of truxenyl anions in specific time windows (0, 1 ps, and 1 ns), and the bottom contour plots present the full timescale up to 7.3 ns. In the fs-TA spectra of **1** (Fig. S22a), because of the near UV band absorption of truxene, only a broad signal around 550 nm is observed, which can be assigned as ESA existing up to 1 ns. In the monoanionic **Na-1^{-}** (Fig. S22b), the first negative signal at 450 nm is correlated to GSB consistent with the absorption maxima in its UV-vis spectrum, followed by the positive ESA signals at 480 and 530 nm. Besides, a long band of stimulated emission (SE) mainly from 560 nm is observed which is close to the photoluminescent result. A positive band over 560 nm can be assigned as additional low-energy ESA. A similar pattern is observed in **Na_2-1^{2-}** (Fig. S22c), where 450 nm and 560 nm correspond to ESA and SE, respectively. In contrast, the three-fold deprotonated **K_3-1^{3-}** behaves differently (Fig. S22d). For example, the GSB comes from 430 nm corresponding to the 426 nm (sh) in the UV-vis spectrum, while a relatively long ESA in the timescale of 100 ps is found at 475 nm. The SE process at 550 is in good agreement with its emission spectrum. Afterward, a positive band (mainly from 650 to 730 nm) can be assigned as additional low-energy ESA. Overall, we used transient absorption spectra to confirm the consistency of the absorption and luminescent results, moreover, some additional excited-state absorption was found for each charging state.

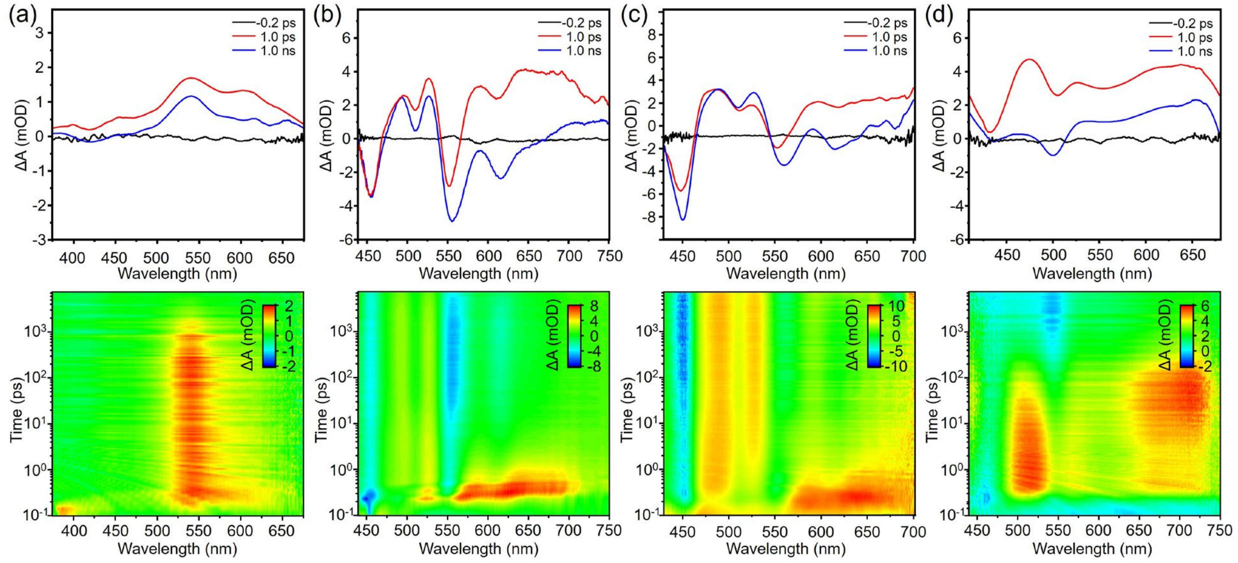

Fig. S22 Femtosecond transient absorption spectra of (a) **1**, (b) **Na-1**$^{-}$, (c) **Na$_2$-1**$^{2-}$, and (d) **K$_3$-1**$^{3-}$ excited at 350 nm in THF at 25 °C.

VII. Crystal Structure Solution and Refinement Details

Data collections of **1**, **Na-1**$^{-}$·2THF, **Na**$_2$**-1**$^{2-}$, and **Cs**$_2$**-1**$^{2-}$ were performed on a Bruker VENTURE system equipped with a PHOTON 100 CMOS detector, a Mo-target fine-focus X-ray source (λ = 0.71073 Å) or a Cu-target microfocus X-ray source (λ = 1.54178 Å), and a graphite monochromator. All data were collected at 100(2) K crystal temperature (Oxford Cryosystems CRYOSTREAM 700) with an appropriate 0.5° ω scan strategy. Data collection of **Cs-1**$^{-}$ and **K**$_3$**-1**$^{3-}$·THF were performed at 100(2) K on a Huber Kappa system with a DECTRIS PILATUS3 X 2M (CdTe) pixel array detector using ϕ scans (synchrotron radiation at λ = 0.41328 Å) located at the Advanced Photon Source, Argonne National Laboratory (NSF's ChemMatCARS, Sector 15, Beamline 15-ID-D). The dataset's reduction and integration were performed with the Bruker software package SAINT (version 8.38A).[2] Data were corrected for absorption effects using the empirical methods as implemented in SADABS (version 2016/2).[3] The structures were solved by SHELXT (version 2018/2)[4] and refined by full-matrix least-squares procedures using the Bruker SHELXTL (version 2019/2)[5] software package through the OLEX2 graphical interface.[6] All non-hydrogen atoms, including those in disordered parts, were refined anisotropically. Hydrogen atoms were included in idealized positions for structure factor calculations with U_{iso}(H) = 1.2 U_{eq}(C) and U_{iso}(H) = 1.5 U_{eq}(C) for methyl groups. The geometries of the disordered parts were restrained to be similar. They were also restrained to have the same U_{ij} components, with a standard uncertainty of 0.01 Å^2. In each unit cell of **Na-1**$^{-}$·2THF, eight THF solvent molecules were found to be severely disordered and removed by the Olex2's solvent mask subroutine.[7] The total void volume was 1,082.6 Å^3, equivalent to 21.42 % of the unit cell's total volume. In each unit cell of **K**$_3$**-1**$^{3-}$·THF, two THF solvent molecules were found to be severely disordered and removed by the Olex2's solvent mask subroutine.[7] The total void volume was 295.8 Å^3, equivalent to 7.74 % of the unit cell's total volume. Further crystal and data collection details are listed in Table S1.

Table S1. Crystal data and structure refinement parameters.

Compound	**1**	**Na-1^{-}**·2THF	**Na$_2$-1^{2-}**
Empirical formula	$C_{27}H_{18}$	$C_{55}H_{73}NaO_{10}$	$C_{67}H_{96}Na_2O_{16}$
Formula weight	342.41	917.12	1203.41
Temperature (K)	100(2)	100(2)	100(2)
Wavelength (Å)	1.54178	1.54178	1.54178
Crystal system	Monoclinic	Orthorhombic	Orthorhombic
Space group	$P2_1$	$Pnna$	$P2_12_12_1$
a (Å)	4.68560(10)	16.8733(6)	13.6941(2)
b (Å)	16.2628(5)	15.5281(6)	21.0294(3)
c (Å)	11.3085(4)	19.2855(7)	22.7326(2)
α (°)	90.00	90.00	90.00
β (°)	90.473(2)	90.00	90.00
γ (°)	90.00	90.00	90.00
V (Å^3)	861.69(4)	5053.0(3)	6546.51(15)
Z	2	4	4
ρ_{calcd} (g·cm^{-3})	1.320	1.206	1.221
μ (mm^{-1})	0.566	0.726	0.808
F(000)	360	1976	2592
Crystal size (mm)	0.06×0.11×0.12	0.05×0.09×0.16	0.10×0.20×0.30
θ range for data collection (°)	9.470-79.599	3.654-63.783	2.862-63.802
Reflections collected	7043	16983	27814
Independent reflections	3143 [R_{int} = 0.0305]	4152 [R_{int} = 0.0499]	10347 [R_{int} = 0.0904]
Transmission factors (min/max)	0.8214/0.9562	0.5938/0.7524	0.8524/0.9635
Data/restraints/params.	3143/1159/488	4152/622/492	10347/812/743
$R1$,[a] $wR2$[b] ($I > 2\sigma(I)$)	0.0405, 0.1050	0.0738, 0.2201	0.1750, 0.3973
$R1$,[a] $wR2$[b] (all data)	0.0439, 0.1076	0.1277, 0.2714	0.2595, 0.4689
Quality-of-fit[c]	1.054	1.040	1.278

$R_{int} = \Sigma|F_o^2 - \langle F_o^2\rangle|/\Sigma|F_o^2|$

[a]$R1 = \Sigma||F_o|-|F_c||/\Sigma|F_o|$. [b]$wR2 = [\Sigma[w(F_o^2-F_c^2)^2]/\Sigma[w(F_o^2)^2]]$.

[c]Quality-of-fit = $[\Sigma[w(F_o^2-F_c^2)^2]/(N_{obs}-N_{params})]^{½}$, based on all data.

Compound	**Cs-1**$^{-}$	**Cs$_2$-1**$^{2-}$	**K$_3$-1**$^{3-}$·THF
Empirical formula	$C_{39}H_{41}CsO_6$	$C_{51}H_{64}Cs_2O_{12}$	$C_{75}H_{111}K_3O_{21}$
Formula weight	738.63	1134.84	1465.93
Temperature (K)	100(2)	100(2)	100(2)
Wavelength (Å)	0.41328	0.71073	0.41328
Crystal system	Monoclinic	Monoclinic	Monoclinic
Space group	*Cc*	$P2_1/c$	$P2_1$
a (Å)	20.9133(9)	21.0136(10)	13.4467(7)
b (Å)	9.6671(3)	12.7649(6)	15.8867(9)
c (Å)	17.8849(7)	21.1357(10)	18.3239(10)
α (°)	90.00	90.00	90.00
β (°)	112.3751(12)	118.744(2)	102.3860(10)
γ (°)	90.00	90.00	90.00
V (Å^3)	3343.6(2)	4970.8(4)	3823.3(4)
Z	4	4	2
ρ_{calcd} (g·cm^{-3})	1.467	1.516	1.273
μ (mm^{-1})	0.285	1.527	0.069
F(000)	1512	2304	1572
Crystal size (mm)	0.11×0.12×0.14	0.04×0.06×0.12	0.09×0.12×0.16
θ range for data collection (°)	1.225-18.631	2.897-28.289	0.902-16.694
Reflections collected	56811	137974	139675
Independent reflections	12298 [R_{int} = 0.0405]	12308 [R_{int} = 0.0561]	21383 [R_{int} = 0.0588]
Transmission factors (min/max)	0.6682/0.7341	0.6168/0.6987	0.5724/0.7440
Data/restraints/params.	12298/1580/660	12308/3277/1156	21383/4175/1673
$R1$,[a] $wR2$[b] ($I > 2\sigma(I)$)	0.0266, 0.0542	0.0410, 0.0731	0.0838, 0.2536
$R1$,[a] $wR2$[b] (all data)	0.0294, 0.0548	0.0492, 0.0751	0.0953, 0.2668
Quality-of-fit[c]	1.132	1.334	1.043

$R_{int} = \Sigma|F_o^2 - \langle F_o^2\rangle|/\Sigma|F_o^2|$

[a] $R1 = \Sigma||F_o|-|F_c||/\Sigma|F_o|$. [b] $wR2 = [\Sigma[w(F_o^2-F_c^2)^2]/\Sigma[w(F_o^2)^2]]$.

[c] Quality-of-fit = $[\Sigma[w(F_o^2-F_c^2)^2]/(N_{obs}-N_{params})]^{1/2}$, based on all data.

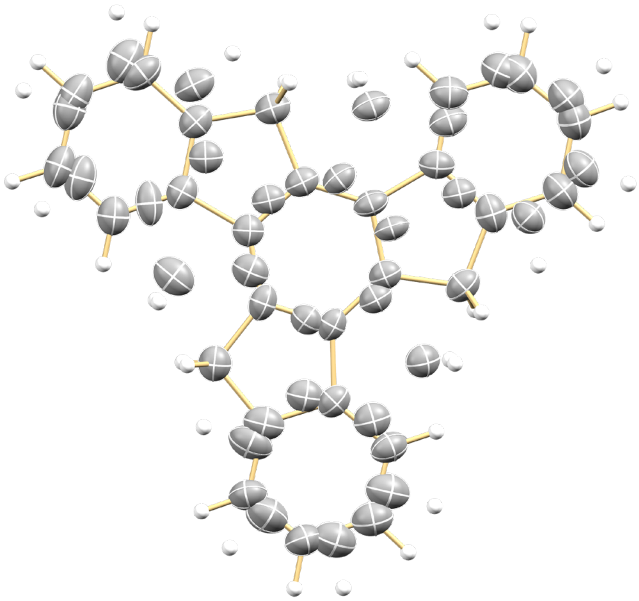

Fig. S23 ORTEP drawing of the asymmetric unit of **1** with two disordered parts, drawn with thermal ellipsoids at the 40% probability level (part 1: 58%, part 2: 42%). The color scheme used: C grey, H white.

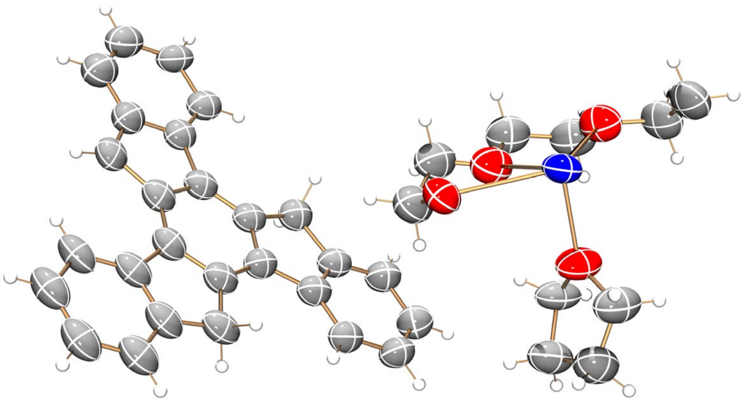

Fig. S24 ORTEP drawing of the asymmetric unit of **Na-1**$^{-}$, drawn with thermal ellipsoids at the 40% probability level. The color scheme used: C grey, H white, O red, Na blue.

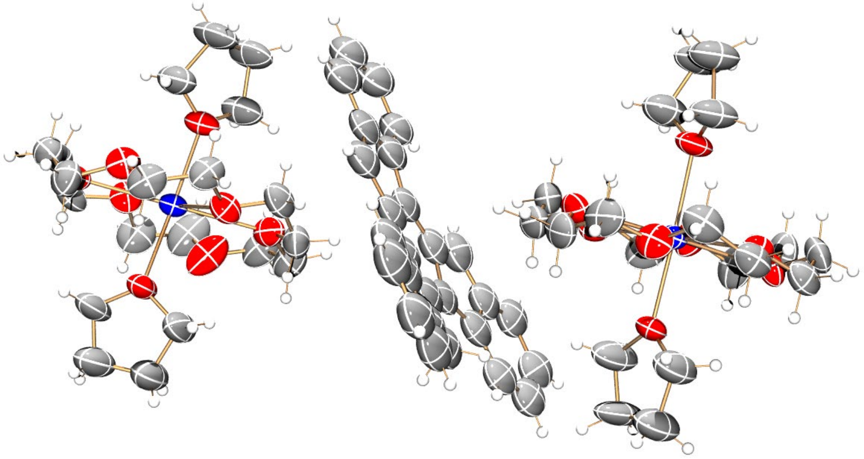

Fig. S25 ORTEP drawing of the asymmetric unit of **Na$_2$-1**$^{2-}$, drawn with thermal ellipsoids at the 40% probability level. The color scheme used: C grey, H white, O red, Na blue.

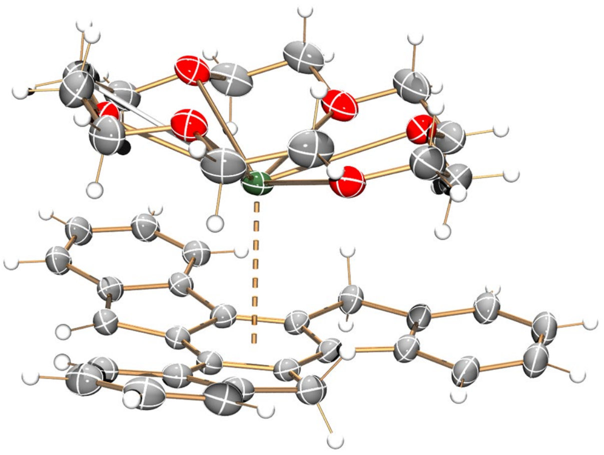

Fig. S26 ORTEP drawing of the asymmetric unit of **Cs-1**$^{-}$, drawn with thermal ellipsoids at the 40% probability level. The color scheme used: C grey, O red, H white, Cs dark green.

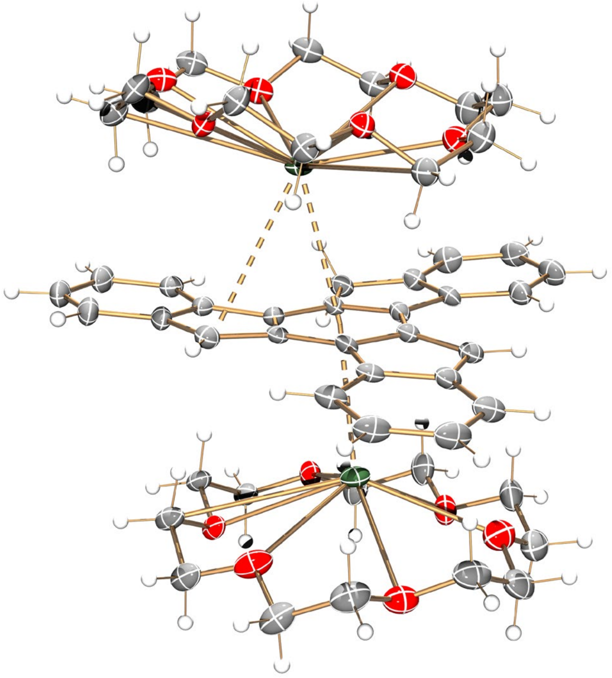

Fig. S27 ORTEP drawing of the asymmetric unit of **Cs$_2$-1**$^{2-}$, drawn with thermal ellipsoids at the 40% probability level. The color scheme used: C grey, O red, H white, Cs dark green.

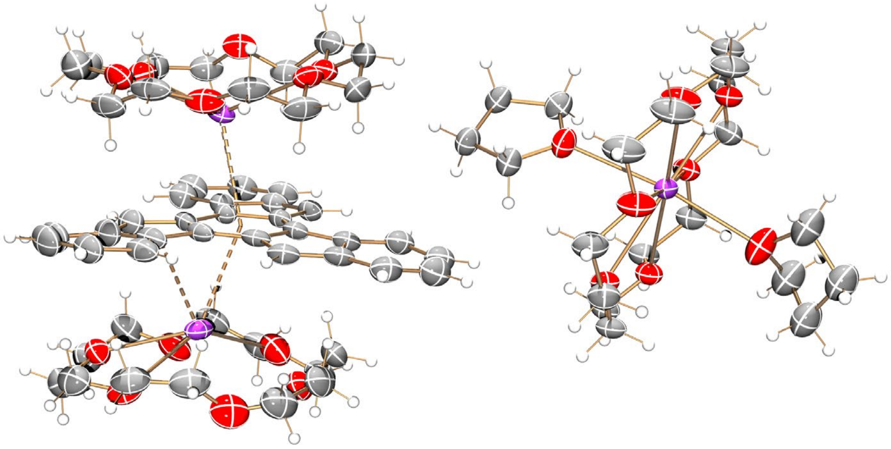

Fig. S28 ORTEP drawing of the asymmetric unit of **K_3-1^{3-}**, drawn with thermal ellipsoids at the 40% probability level. The color scheme used: C grey, O red, H white, K purple.

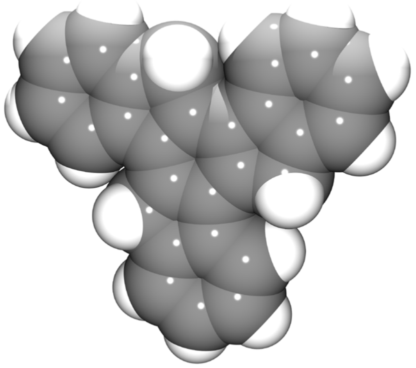

Fig. S29 Crystal structure of **1**, space-filling model.

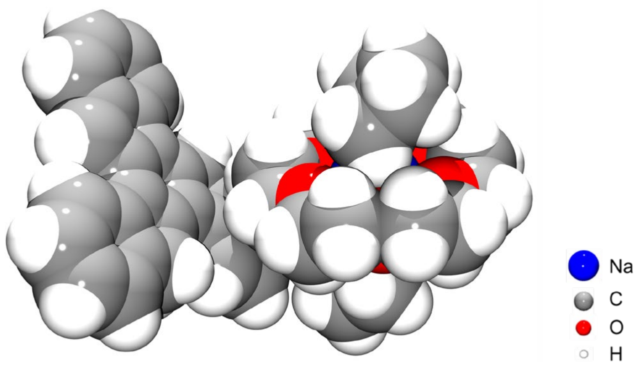

Fig. S30 Crystal structure of **Na-1^{-}**, space-filling model.

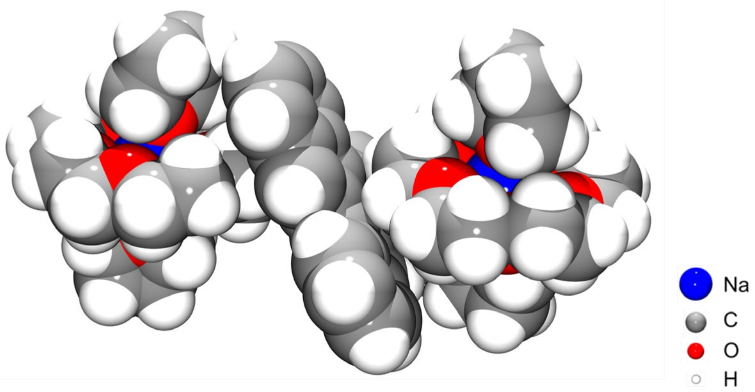

Fig. S31 Crystal structure of **Na_2-1^{2-}**, space-filling model.

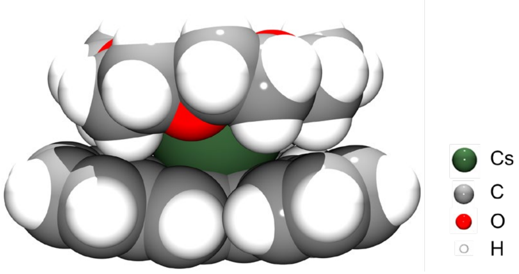

Fig. S32 Crystal structure of **Cs-1^{-}**, space-filling model.

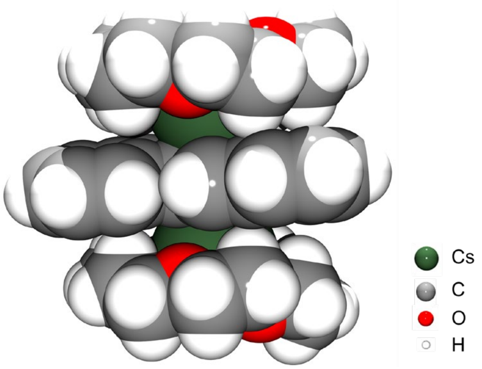

Fig. S33 Crystal structure of **Cs_2-1^{2-}**, space-filling model.

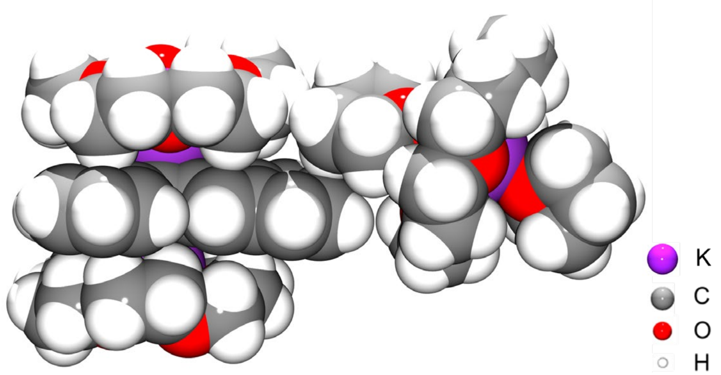

Fig. S34 Crystal structure of $\mathbf{K_3\text{-}1^{3-}}$, space-filling model.

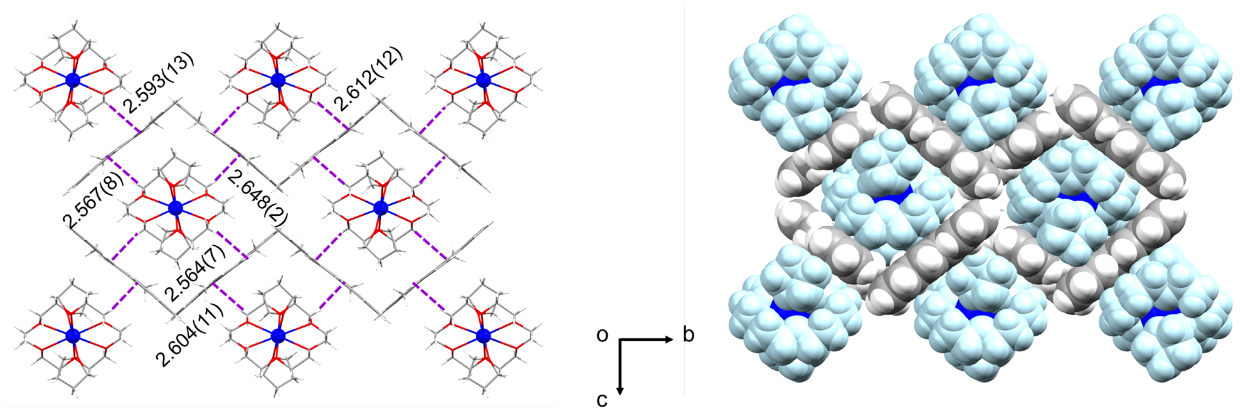

Fig. S35 C–H···π interactions in capped-stick model and solid-state packing of $\mathbf{Na\text{-}1^{-}}$ in space-filling model. {Na^+(18-crown-6)(THF)$_2$} moieties are shown in different shades of blue. Color scheme used: C gray, O red, Na blue, H white.

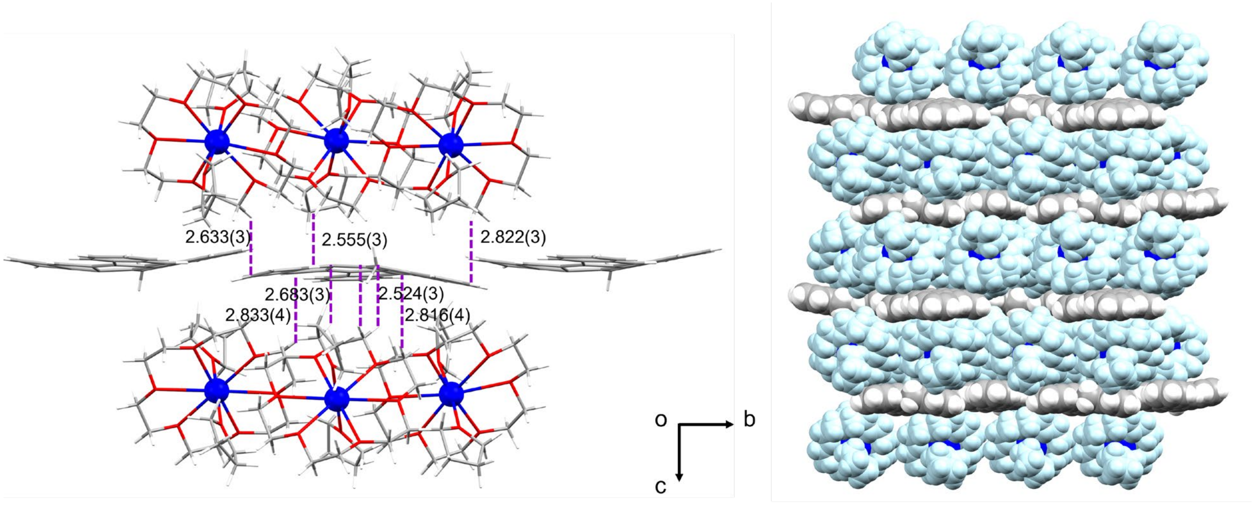

Fig. S36 C–H···π interactions in capped-stick model and solid-state packing of **Na_2-1^{2-}** in space-filling model. {Na^+(18-crown-6)$(THF)_2$} moieties are shown in different shades of blue. Color scheme used: C gray, O red, Na blue, H white.

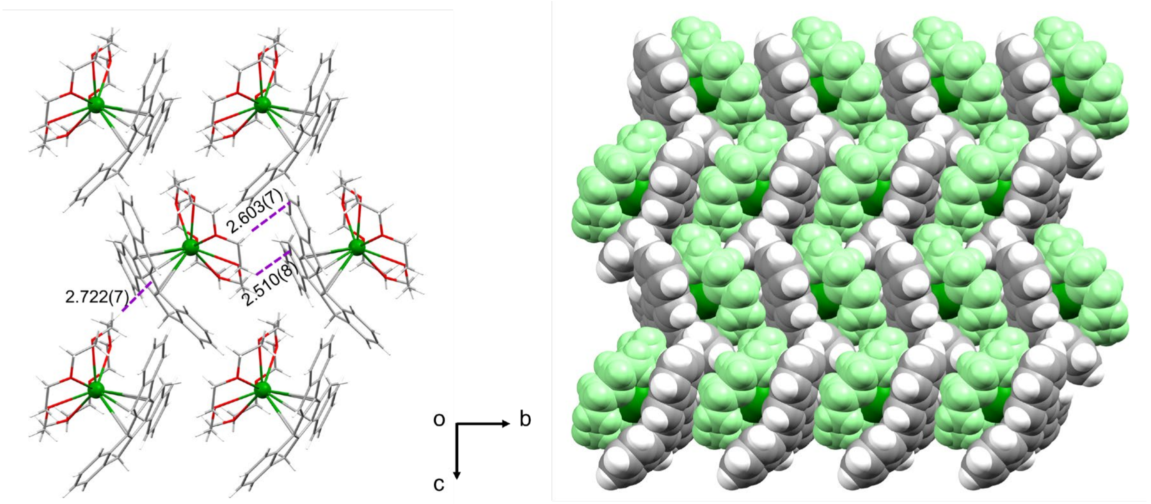

Fig. S37 C–H···π interactions in capped-stick model and solid-state packing of **Cs-1^-** in space-filling model. {Cs^+(18-crown-6)} moieties are shown in different shades of green. Color scheme used: C gray, O red, Cs dark green, H white.

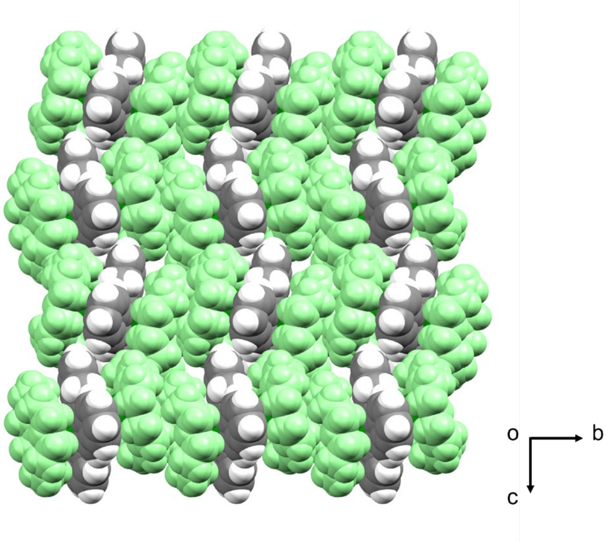

Fig. S38 C–H···π interactions in capped-stick model and solid-state packing of **Cs_2-1^{2-}** in space-filling model. {Cs^+(18-crown-6)} moieties are shown in different shades of green. Color scheme used: C gray, O red, Cs dark green, H white. No significant interactions are found between adjacent molecules.

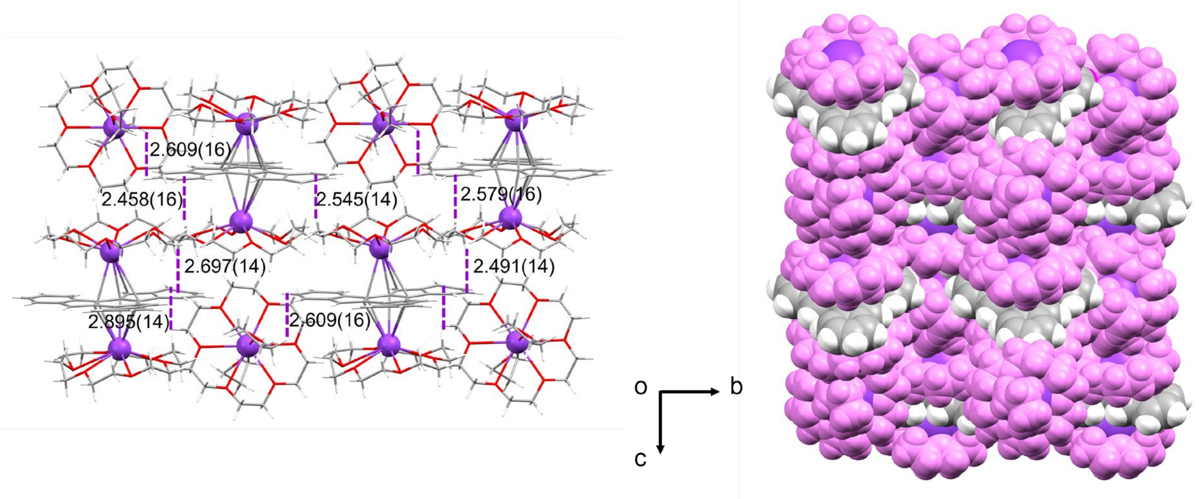

Fig. S39 C–H···π interactions in capped-stick model and solid-state packing of **K_3-1^{3-}** in space-filling model. {K^+(18-crown-6)$(THF)_x$} moieties are shown in different shades of purple. Color scheme used: C gray, O red, K dark orchid, H white.

Table S2. Selected C–C bond length distances (Å) along with a labeling scheme.

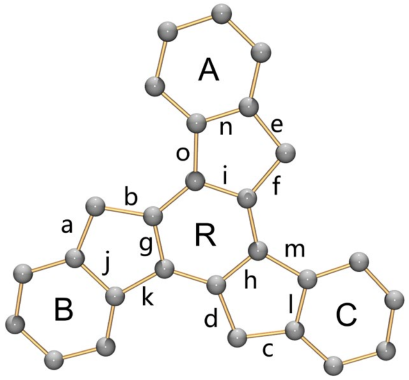

Bond	**1**	**Na-1**$^{-}$	**Na**$_2$**-1**$^{2-}$	**Cs-1**$^{-}$	**Cs**$_2$**-1**$^{2-}$	**K**$_3$**-1**$^{3-}$
a	1.490(10)	1.438(14)	1.51(2)	1.426(6)	1.416(8)	1.388(18)
b	1.488(7)	1.448(13)	1.55(3)	1.431(5)	1.431(7)	1.410(2)
c	1.511(8)	1.513(14)	1.51(3)	1.507(6)	1.416(7)	1.422(15)
d	1.501(6)	1.460(11)	1.57(3)	1.513(7)	1.489(7)	1.399(18)
e	1.400(8)	1.364(15)	1.41(3)	1.398(7)	1.440(8)	1.440(2)
f	1.406(7)	1.433(15)	1.42(3)	1.446(6)	1.423(8)	1.403(2)
g	1.476(8)	1.487(15)	1.43(2)	1.441(6)	1.447(8)	1.451(2)
h	1.401(8)	1.402(19)	1.44(3)	1.400(7)	1.441(8)	1.429(17)
i	1.390(7)	1.412(16)	1.36(3)	1.413(6)	1.415(8)	1.456(3)
j	1.516(6)	1.482(14)	1.54(3)	1.499(6)	1.485(7)	1.437(18)
k	1.412(10)	1.402(16)	1.39(2)	1.436(7)	1.419(8)	1.378(19)
l	1.492(7)	1.514(14)	1.44(3)	1.516(6)	1.422(8)	1.355(2)
m	1.475(7)	1.460(11)	1.45(3)	1.461(6)	1.437(8)	1.450(2)
n	1.377(9)	1.390(1)	1.39(3)	1.407(7)	1.413(8)	1.460(4)
o	1.476(8)	1.449(13)	1.46(3)	1.461(6)	1.465(7)	1.402(14)

VIII. Computational Details

All calculations were performed with Gaussian 16 suite of programs, revision A.03.[8] The geometries of all structures (neutral truxene, monoanion, dianion, and trianion) were optimized using B3LYP[9, 10] functional in conjunction with def2-TZVP[11] basis set, and frequencies analysis was performed to ensure true minima. Dispersion effects were accounted for using Grimme's D3 correction with Becke−Johnson damping.[12, 13] The Aroma program was utilized for generating NICS-Scan input files,[14] and NICS values (reported in ppm) were calculated at the same level using the gauge-including atomic orbital (GIAO) method. The partial charges discussed in the text are Loewdin charges calculated for the carbon atoms with the bonded hydrogens. To obtain partial charges for each ring, the values were summed over all carbons within each ring. ACID plots[15] were generated with the Gaussian 09 suite of programs, revision D.01.[16] The electrostatic potential (ESP) maps and ACID plots for the corresponding structures were generated by GaussView 6.0. MO diagrams were generated by IboView.

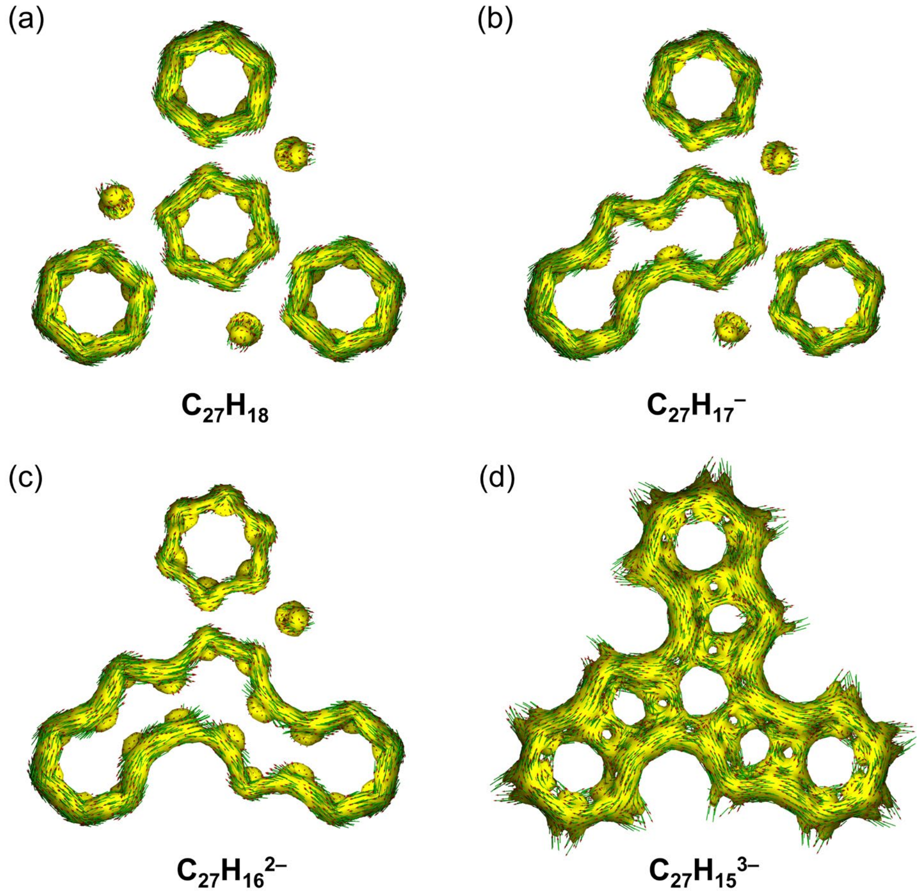

Fig. S40 π-orbital ACID isosurfaces for (a) $C_{27}H_{18}$, (b) $C_{27}H_{17}^{-}$, (c) $C_{27}H_{16}^{2-}$, and (d) $C_{27}H_{15}^{3-}$.

Nature of transitions in $C_{27}H_{18}$

***S*₁: 320 nm** (f = 0.000)

HOMO–1 → LUMO	(12.7%)
HOMO–1 → LUMO+1	(36.5%)
HOMO → LUMO	(36.5%)
HOMO → LUMO+1	(12.7%)

***S*₂: 282 nm** (f = 0.754)

HOMO–1 → LUMO	(12.7%)
HOMO–1 → LUMO+1	(16.8%)
HOMO–1 → LUMO+2	(3.3%)
HOMO → LUMO	(27.7%)
HOMO → LUMO+1	(31.1%)
HOMO → LUMO+2	(1.3%)

***S*₃: 282 nm** (f = 0.751)

HOMO–1 → LUMO	(35%)
HOMO–1 → LUMO+1	(14.2%)
HOMO–1 → LUMO+4	(1.1%)
HOMO → LUMO	(25.4%)
HOMO → LUMO+1	(13.8%)
HOMO → LUMO+4	(3.1%)

***S*₄: 282 nm** (f = 0.049)

HOMO–1 → LUMO	(31.5%)
HOMO–1 → LUMO+1	(24.7%)
HOMO → LUMO	(2.6%)

***S*₅: 266 nm** (f = 0.158**)**

HOMO–5 → LUMO+1	(4.1%)
HOMO–4 → LUMO+1	(3.3%)
HOMO–4 → LUMO+3	(2.0%)
HOMO–3 → LUMO	(3.3%)
HOMO–2 → LUMO+1	(3.4%)
HOMO–1 → LUMO	(2.7%)
HOMO–1 → LUMO+2	(44.9%)
HOMO–1 → LUMO+3	(1.1%)
HOMO → LUMO+1	(2.6%)
HOMO → LUMO+2	(27.0%)

***S*₆: 266 nm** (f = 0.157)

HOMO–5 → LUMO	(4.1%)
HOMO–4 → LUMO	(3.3%)
HOMO–3 → LUMO+1	(3.3%)
HOMO–3 → LUMO+3	(2.0%)
HOMO–2 → LUMO	(3.3%)
HOMO–1 → LUMO+1	(2.6%)
HOMO–1 → LUMO+2	(27.0%)
HOMO → LUMO	(2.6%)
HOMO → LUMO+2	(44.9%)
HOMO → LUMO+3	(1.1%)

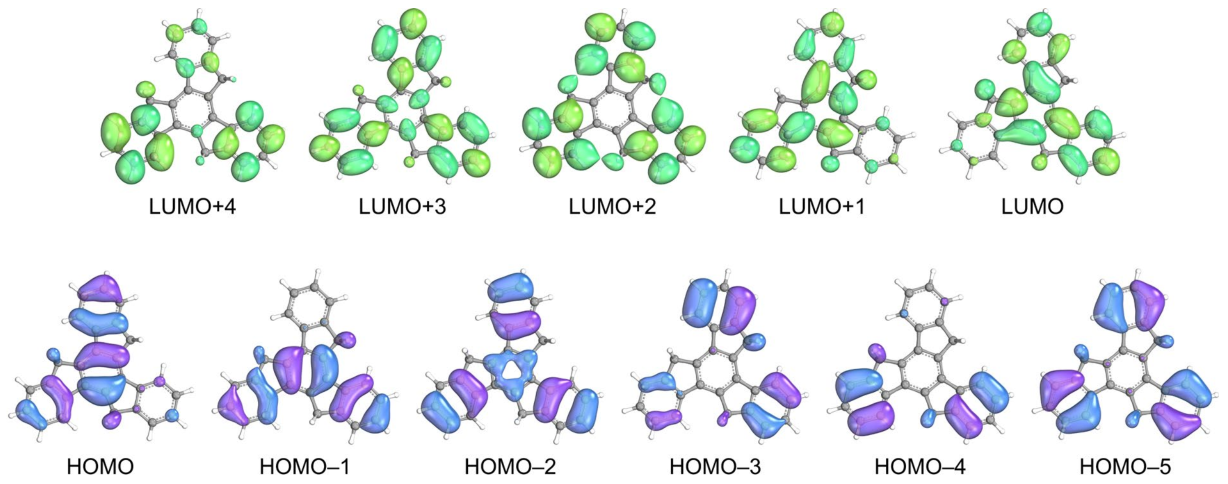

Fig. S41. Active MOs in $C_{27}H_{18}$ (B3LYP/def2-ZVP/CPCM(THF)).

Nature of transitions in $C_{27}H_{17}^{-}$

***S*₁: 482 nm** (f = 0.205)

HOMO → LUMO	(95.1%)
HOMO–1 → LUMO+1	(1.0%)

***S*₂: 422 nm** (f = 0.292)

HOMO–1 → LUMO	(2.6%)
HOMO → LUMO	(1.3%)
HOMO → LUMO+1	(74.5%)
HOMO → LUMO+2	(19.7%)

***S*₃: 399 nm** (f = 0.353)

HOMO–1 → LUMO	(2.7%)
HOMO → LUMO+1	(44.9%)
HOMO → LUMO+2	(1.1%)

***S*₄: 383 nm** (f = 0.121)

HOMO → LUMO+3	(97.5%)

***S*₅: 374 nm** (f = 0.051)

HOMO → LUMO+4	(95.8%)

***S*₆: 314 nm** (f = 0.524)

HOMO–2 → LUMO	(4.5%)
HOMO–1 → LUMO	(80.7%)
HOMO–1 → LUMO+1	(4.1%)
HOMO → LUMO+1	(3.4%)
HOMO → LUMO+4	(1.0%)

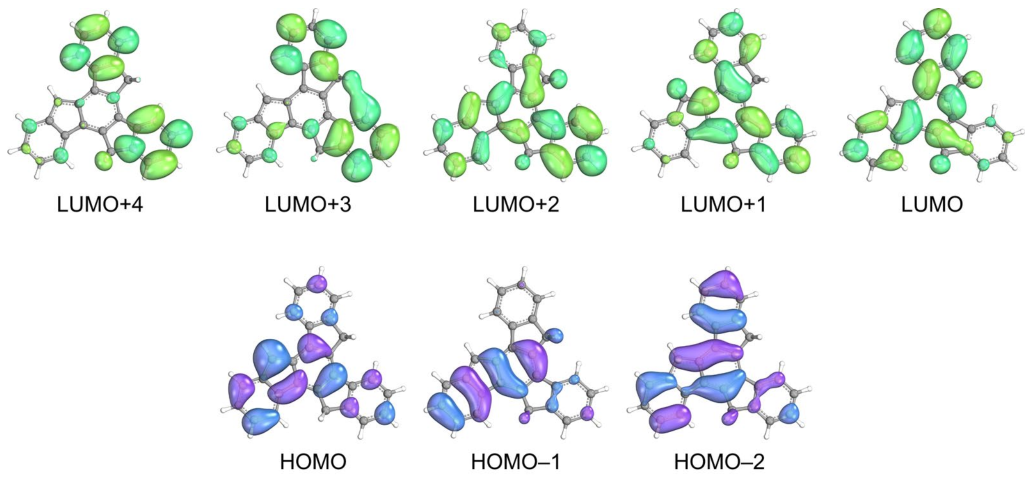

Fig. S42. Active MOs in $C_{27}H_{17}^{-}$ (B3LYP/def2-TZVP/CPCM(THF)).

Nature of transitions in $C_{27}H_{16}^{2-}$

***S*$_1$: 483 nm** (f = 0.048)

HOMO–1 → LUMO (54.1%)

HOMO → LUMO (38.5%)

HOMO → LUMO+1 (4.2%)

***S*$_2$: 462 nm** (f = 0.160)

HOMO–1 → LUMO (37.9%)

HOMO–1 → LUMO+1 (6.8%)

HOMO–1 → LUMO+2 (5.3%)

HOMO → LUMO (37.8%)

HOMO → LUMO+1 (6.6%)

HOMO → LUMO+2 (3.1%)

***S*$_3$: 431 nm** (f = 0.109)

HOMO–1 → LUMO+1 (24.3%)

HOMO–1 → LUMO+2 (2.2%)

HOMO–1 → LUMO+3 (1.3%)

HOMO → LUMO (2.3%)

HOMO → LUMO+1 (64.3%)

HOMO → LUMO+2 (3.4%)

***S*$_4$: 416 nm** (f = 0.313)

HOMO–1 → LUMO+1 (65.0%)

HOMO–1 → LUMO+2 (2.7%)

HOMO → LUMO (10.3%)

HOMO → LUMO+1 (17.2%)

HOMO → LUMO+3 (1.4%)

***S*$_5$: 410 nm** (f = 0.091)

HOMO–1 → LUMO (1.3%)

HOMO–1 → LUMO+2 (14.2%)

HOMO–1 → LUMO+3 (17.0%)

HOMO → LUMO+1 (1.3%)

HOMO → LUMO+2 (61.6%)

***S*$_6$: 393 nm** (f = 0.186)

HOMO–1 → LUMO (1.6%)

HOMO–1 → LUMO+2 (54.9%)

HOMO–1 → LUMO+3 (18.8%)

HOMO → LUMO (2.0%)

HOMO → LUMO+2 (3.4%)

HOMO → LUMO+3 (14.7%)

***S*$_7$: 384 nm** (f = 0.214)

HOMO–1 → LUMO+2 (9.6%)

HOMO–1 → LUMO+3 (2.4%)

HOMO → LUMO (3.2%)

HOMO → LUMO+1 (2.3%)

HOMO → LUMO+3 (79.4%)

***S*₈: 356 nm** (f = 1.165)

HOMO–1 → LUMO	(2.3%)
HOMO–1 → LUMO+1	(1.4%)
HOMO–1 → LUMO+2	(7.4%)
HOMO–1 → LUMO+3	(55.5%)
HOMO–1 → LUMO+5	(1.0%)
HOMO → LUMO	(2.7%)
HOMO → LUMO+1	(1.5%)
HOMO → LUMO+2	(23.5%)
HOMO → LUMO+5	(1.1%)

***S*₉: 305 nm** (f = 0.181)

HOMO–2 → LUMO	(86.4%)
HOMO–1 → LUMO+4	(1.8%)
HOMO–1 → LUMO+5	(4.3%)
HOMO → LUMO+4	(1.5%)
HOMO → LUMO+5	(1.6%)

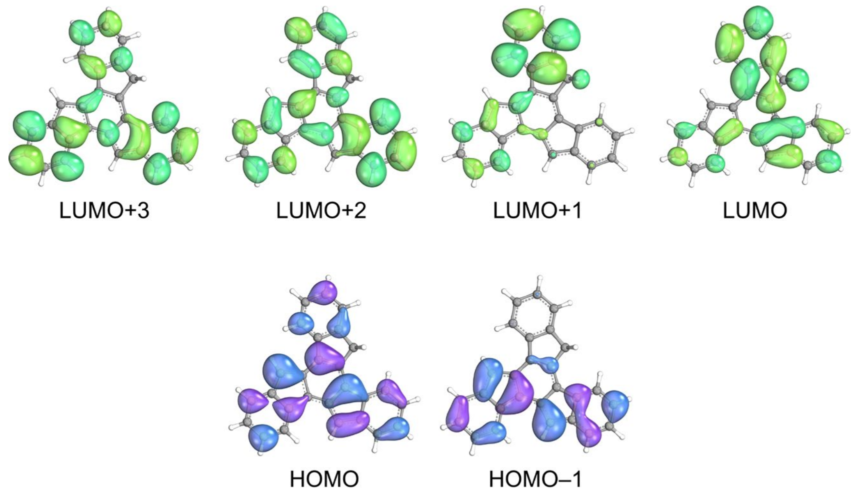

Fig. S43. Active MOs in $C_{27}H_{16}^{2-}$ (B3LYP/def2-TZVP/CPCM(THF)).

Nature of transitions in $C_{27}H_{15}^{3-}$

***S*$_1$: 445 nm** (f = 0.000)

HOMO–2 → LUMO+2	(2.6%)
HOMO–1 → LUMO	(47.8%)
HOMO → LUMO+1	(48.0%)

***S*$_2$: 435 nm** (f = 0.000)

HOMO–1 → LUMO+1	(48.4%)
HOMO → LUMO	(48.7%)

***S*$_3$: 416 nm** (f = 0.037)

HOMO–2 → LUMO	(6.7%)
HOMO–1 → LUMO	(10.5%)
HOMO–1 → LUMO+1	(5.8%)
HOMO–1 → LUMO+2	(24.6%)
HOMO → LUMO	(5.7%)
HOMO → LUMO+1	(10.3%)
HOMO → LUMO+2	(31.3%)

***S*$_4$: 416 nm** (f = 0.038)

HOMO–2 → LUMO+1	(6.6%)
HOMO–1 → LUMO	(16.0%)
HOMO–1 → LUMO+1	(10.3%)
HOMO–1 → LUMO+2	(31.0%)
HOMO → LUMO	(10.1%)
HOMO → LUMO+1	(5.9%)
HOMO → LUMO+2	(25.1%)

***S*$_5$: 393 nm** (f = 0.249)

HOMO–2 → LUMO	(21.9%)
HOMO–2 → LUMO+1	(17.2%)
HOMO–1 → LUMO+1	(14.5%)
HOMO–1 → LUMO+2	(4.9%)
HOMO → LUMO	(14.5%)
HOMO → LUMO+2	(22.8%)

***S*$_6$: 393 nm** (f = 0.248)

HOMO–2 → LUMO	(17.3%)
HOMO–2 → LUMO+1	(21.9%)
HOMO–1 → LUMO	(14.2%)
HOMO–1 → LUMO+2	(23.3%)
HOMO → LUMO+1	(14.3%)
HOMO → LUMO+2	(5.0%)

***S*$_7$: 363 nm** (f = 0.001)

HOMO–2 → LUMO+2	(95.3%)
HOMO–1 → LUMO	(1.7%)
HOMO → LUMO+1	(1.1%)

S_8: 363 nm (f = 1.009)

HOMO–2 → LUMO	(49.0%)
HOMO–1 → LUMO	(10.1%)
HOMO–1 → LUMO+1	(5.1%)
HOMO–1 → LUMO+2	(2.4%)
HOMO → LUMO	(5.1%)
HOMO → LUMO+1	(10.7%)
HOMO → LUMO+2	(8.4%)
HOMO → LUMO+3	(4.4%)

S_9: 362 nm (f = 1.011)

HOMO–2 → LUMO+1	(49.0%)
HOMO–1 → LUMO	(5.0%)
HOMO–1 → LUMO+1	(10.4%)
HOMO–1 → LUMO+2	(8.6%)
HOMO–1 → LUMO+3	(4.4%)
HOMO → LUMO	(10.5%)
HOMO → LUMO+1	(5.0%)
HOMO → LUMO+2	(2.3%)

S_{10}: 313 nm (f = 0.323)

HOMO–2 → LUMO	(1.7%)
HOMO–1 → LUMO+2	(2.5%)
HOMO → LUMO+3	(89.1%)

S_{11}: 312 nm (f = 0.323)

HOMO–2 → LUMO+1	(41.7%)
HOMO–1 → LUMO+3	(89.1%)
HOMO → LUMO+2	(2.4%)

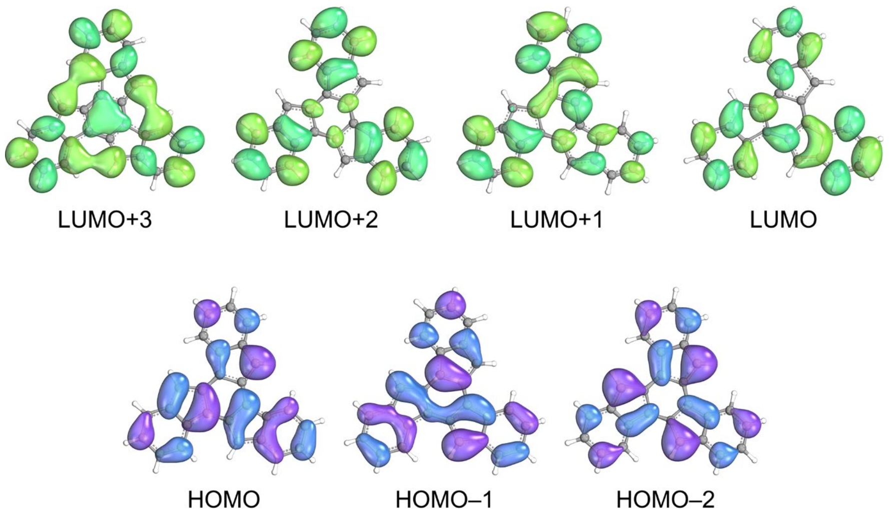

Fig. S44. Active MOs in $C_{27}H_{15}^{3-}$ (B3LYP/def2-TZVP/CPCM(THF)).

Table S3. Cartesian coordinates of the $C_{27}H_{18}$.

Total Energy: -1040.173001 Hartree

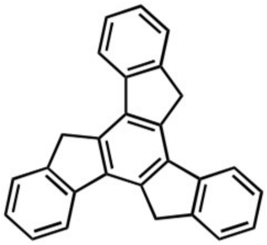

Atomic Number	X	Y	Z
6	-3.97037	-3.3859	-0.000215
1	-4.21375	-4.44105	-0.000414
6	-4.99578	-2.4428	-0.000254
1	-6.02755	-2.77088	-0.000395
6	4.613506	-3.1049	0.000192
1	5.413555	-3.83436	0.000222
6	0.250589	-1.37646	0.000139
6	-3.37754	-0.67779	0.000226
6	0.382224	5.547784	0.000343
1	0.613933	6.605376	0.000636
6	-1.31731	0.471167	-0.000031
6	1.415803	4.61132	0.000004
1	2.449031	4.938308	-0.000031
6	-1.05864	-0.91533	0.000114
6	1.066761	0.905212	-0.000263
6	3.285709	-3.53176	0.000324
1	3.052272	-4.59006	0.000586
6	-0.26331	1.374409	-0.000212
6	2.578165	-1.21288	-0.000028
6	-2.33949	-1.62629	0.000211
6	1.321977	-0.45918	-0.000062
6	-0.94722	5.131261	0.000349
1	-1.73935	5.869571	0.000613
6	1.101773	3.2639	-0.000349
6	-4.70147	-1.07949	-0.000062
1	-5.50122	-0.34813	-0.000162
6	2.27581	-2.58611	0.0002
6	-1.26861	3.777035	0.00006
1	-2.30432	3.466271	0.000063
6	-2.63686	-2.98719	-0.000012
1	-1.84996	-3.72886	-0.000013
6	4.917501	-1.7453	-0.000062
1	5.952955	-1.42843	-0.000139
6	-0.23866	2.83914	-0.000256
1	0.445885	-3.35043	-0.877892
1	0.445614	-3.35089	0.877691
6	3.905395	-0.78986	-0.000176
1	4.154168	0.262467	-0.000352
6	0.780563	-2.78814	0.00013
6	-2.80484	0.718117	0.000215

1	-3.12481	1.289419	-0.877425
1	-3.12432	1.289263	0.87815
6	2.024337	2.069991	-0.000494
1	2.678653	2.061209	-0.878458
1	2.679104	2.061372	0.877126

Table S4. Cartesian coordinates of the $C_{27}H_{17}^{-}$.

Total Energy: -1039.613260 Hartree

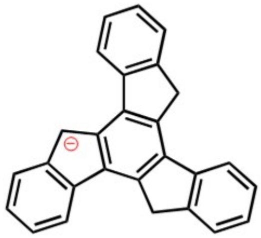

Atomic Number	X	Y	Z
6	0.843376	-5.13587	-0.000379
1	1.626776	-5.8859	-0.000676
6	-0.48951	-5.53649	-0.000365
1	-0.74109	-6.59028	-0.000517
6	5.058126	2.352156	0.000249
1	6.098384	2.655599	0.000315
6	1.298322	-0.46355	0.000045
6	-1.17138	-3.23244	0.000309
6	-4.52653	3.248414	-0.000111
1	-5.30136	4.008221	-0.000155
6	-1.09621	-0.86597	0.000172
6	-3.19515	3.629684	-0.000338
1	-2.9299	4.682315	-0.000568
6	0.226973	-1.36562	0.00016
6	-0.24525	1.444118	-0.000141
6	4.723979	0.992553	0.000215
1	5.504494	0.238569	0.000432
6	-1.35246	0.491743	0.000028
6	2.366382	1.592425	-0.000073
6	0.181121	-2.81639	0.000197
6	1.075848	0.917617	-0.000083
6	-4.89451	1.890146	0.000196
1	-5.9434	1.616345	0.000379
6	-2.18582	2.649882	-0.000267
6	-1.50505	-4.5707	-0.000019
1	-2.54642	-4.87598	-0.000064
6	3.393762	0.623865	0.000044
6	-3.91738	0.902099	0.000279
1	-4.20818	-0.1423	0.000489
6	1.19011	-3.78529	-0.000147
1	2.232662	-3.49594	-0.000258
6	4.050836	3.313343	0.000069
1	4.31637	4.364756	0.00008

6	-2.56731	1.258677	0.000025
6	-2.07162	-2.01939	0.000491
1	-2.72813	-1.99714	-0.876092
6	2.779941	-0.75353	-0.000075
1	3.086766	-1.33347	-0.878251
1	3.087137	-1.3341	0.877494
6	-0.76781	2.743202	-0.000356
1	-0.21092	3.666875	-0.000555
6	2.705387	2.947667	-0.000061
1	1.935095	3.705237	-0.000085
1	-2.72738	-1.9969	0.877643

Table S5. Cartesian coordinates of the $C_{27}H_{16}{}^{2-}$.

Total Energy: -1038.938255 Hartree

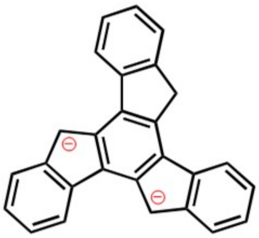

Atomic Number	X	Y	Z
6	4.424821	-2.81044	-0.000001
1	5.508235	-2.73776	-0.000009
6	3.805529	-4.0709	-0.000045
1	4.418814	-4.96865	-0.000029
6	1.589834	5.338007	-0.000058
1	2.052105	6.319217	-0.000087
6	1.213508	0.63869	0.000103
6	1.609629	-3.03551	-0.000049
6	-5.45891	-1.23276	0.000071
1	-6.54388	-1.30366	0.00013
6	-0.06556	-1.4637	-0.000079
6	-4.84966	0.014617	0.000129
1	-5.45887	0.916192	0.000203
6	1.20299	-0.75162	0.000021
6	-1.24335	0.73217	0.000057
6	2.384351	4.175242	0.000095
1	3.468291	4.256184	0.000196
6	-1.27306	-0.71085	-0.000056
6	0.368856	2.794041	0.000005
6	2.253928	-1.73219	0
6	0.025533	1.394524	0.000056
6	-4.68505	-2.41181	-0.000036
1	-5.17807	-3.37973	-0.00011
6	-3.4459	0.119923	0.000078
6	2.418133	-4.1864	-0.000053
1	1.956693	-5.17103	-0.000094
6	1.783433	2.936963	0.000111

6	-3.29438	-2.34087	-0.000082
1	-2.70973	-3.2536	-0.000198
6	3.645643	-1.64856	0.000001
1	4.133027	-0.67893	0.000038
6	0.202464	5.210165	-0.000239
1	-0.41395	6.105032	-0.000402
6	-2.64701	-1.09942	-0.000006
6	0.205307	-2.84684	-0.000087
1	-0.52645	-3.64118	-0.000113
6	2.409132	1.563297	0.000163
1	3.048375	1.40584	-0.876943
1	3.048308	1.405827	0.877313
6	-2.56852	1.226269	0.000093
1	-2.87322	2.262504	0.000639
6	-0.41738	3.960528	-0.000201
1	-1.49572	3.888396	-0.000474

Table S6. Cartesian coordinates of the $C_{27}H_5^{3-}$.

Total Energy: -1038.156259 Hartree

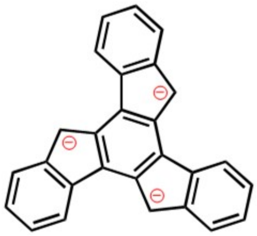

Atomic Number	X	Y	Z
6	2.123882	4.813989	-4E-06
1	1.890674	5.877341	-1.4E-05
6	3.468102	4.390673	-1E-06
1	4.268648	5.129939	0.000004
6	-5.53655	0.808059	0.00001
1	-6.57705	1.131714	0.000004
6	-0.87633	1.141402	-9E-06
6	2.760151	2.058754	-5E-06
6	2.068574	-5.1987	-6E-06
1	2.3086	-6.26161	-0.00001
6	1.426629	0.188199	-1.2E-05
6	0.734134	-4.78747	-4.6E-05
1	-0.06069	-5.53388	-8.2E-05
6	0.544104	1.327879	-1.3E-05
6	-0.55037	-1.32963	0.000024
6	-4.51314	1.758026	0.000045
1	-4.76203	2.819594	0.000089
6	0.877878	-1.13516	-6E-06
6	-2.84408	-0.07356	-2.4E-05
6	1.358355	2.499769	-1.4E-05
6	-1.42206	-0.19274	0.000021
6	3.107225	-4.24615	0.000051

1	4.144748	-4.57578	0.000085
6	0.402899	-3.41976	-3.2E-05
6	3.77908	3.029385	0.000006
1	4.822872	2.714151	0
6	-3.16308	1.360922	0.000041
6	2.811668	-2.87703	0.000077
1	3.622966	-2.15691	0.000076
6	1.085879	3.873556	-1.5E-05
1	0.056629	4.216252	-1.8E-05
6	-5.23102	-0.56772	-5.5E-05
1	-6.03528	-1.30139	-0.00007
6	1.485673	-2.42623	0.000033
6	2.770752	0.649583	-6.8E-05
6	-1.94801	2.074709	0.000049
6	-0.82281	-2.72438	0.000019
1	-1.80186	-3.18391	-0.00013
6	-3.89757	-0.99642	-6.8E-05
1	-3.67978	-2.05913	-6.7E-05
1	3.658225	0.031415	0.000016
1	-1.8565	3.152353	0.000114
6	2.123882	4.813989	-4E-06

IX. References

(1) Kozhemyakina, N. V.; Nuss, J.; Jansen, M. Demonstration of the “Break-and-Seal” Approach to Fullerides of Complex Cations at the Example of $KC_{60}(THF)_5 \cdot 2THF$. *Z. Anorg. Allg. Chem.* **2009,** *635*, 1355-1361.
(2) SAINT; part of Bruker APEX3 software package (version 2017.3-0): Bruker AXS **2017**.
(3) SADABS; part of Bruker APEX3 software package (version 2017.3-0): Bruker AXS **2017**.
(4) Sheldrick, G. M. SHELXT - Integrated Space-Group and Crystal-Structure Determination. *Acta Crystallogr.* **2015,** *A71*, 3-8.
(5) Sheldrick, G. M. Crystal Structure Refinement with SHELXL. *Acta Crystallogr.* **2015,** *C71*, 3-8.
(6) Dolomanov, O. V.; Bourhis, L. J.; Gildea, R. J.; Howard, J. A. K.; Puschmann, H. OLEX2: A Complete Structure Solution, Refinement and Analysis Program. *J. Appl. Crystallogr.* **2009,** *42*, 339-341.
(7) Spek, A. L. PLATON SQUEEZE: A Tool for the Calculation of the Disordered Solvent Contribution to the Calculated Structure Factors. *Acta Crystallogr C* **2015,** *71*, 9-18.
(8) M. J. Frisch, G. W. Trucks, H. B. Schlegel, G. E. Scuseria, M. A. Robb, J. R. Cheeseman, G. Scalmani, V. Barone, B. Mennucci, G. A. Petersson, H. Nakatsuji, M. Caricato, X. Li, H. P. Hratchian, A. F. Izmaylov, J. Bloino, G. Zheng, J. L. Sonnenberg, M. Hada, M. Ehara, K. Toyota, R. Fukuda, J. Hasegawa, M. Ishida, T. Nakajima, Y. Honda, O. Kitao, H. Nakai, T. Vreven, J. A. Montgomery, Jr., J. E. Peralta, F. Ogliaro, M. Bearpark, J. J. Heyd, E. Brothers, K. N. Kudin, V. N. Staroverov, R. Kobayashi, J. Normand, K. Raghavachari, A. Rendell, J. C. Burant, S. S. Iyengar, J. Tomasi, M. Cossi, N. Rega, J. M. Millam, M. Klene, J. E. Knox, J. B. Cross, V. Bakken, C. Adamo, J. Jaramillo, R. Gomperts, R. E. Stratmann, O. Yazyev, A. J. Austin, R. Cammi, C. Pomelli, J. W. Ochterski, R. L. Martin, K. Morokuma, V. G. Zakrzewski, G. A. Voth, P. Salvador, J. J. Dannenberg, S. Dapprich, A. D. Daniels, Ö. Farkas, J. B. Foresman, J. V. Ortiz, J. Cioslowski, and D. J. Fox, Gaussian 16, Revision A.03, Gaussian, Inc., Wallingford CT, **2016**.
(9) Lee, C.; Yang, W.; Parr, R. G. Development of the Colle-Salvetti correlation-energy formula into a functional of the electron density. *Phys. Rev. B* **1988,** *37*, 785-789.
(10) Becke, A. D. Density-functional thermochemistry. III. The role of exact exchange. *J. Chem. Phys.* **1993,** *98*, 5648-5652.
(11) Weigend, F.; Ahlrichs, R. Balanced Basis Sets of Split Valence, Triple Zeta Valence and Quadruple Zeta Valence Quality for H to Rn: Design and Assessment of Accuracy. *PCCP* **2005,** *7*, 3297-3305.
(12) Grimme, S.; Antony, J.; Ehrlich, S.; Krieg, H. A Consistent and Accurate *ab* Initio Parametrization of Density Functional Dispersion Correction (DFT-D) for the 94 Elements H-Pu. *J. Chem. Phys.* **2010,** *132*, 154104.
(13) Grimme, S.; Ehrlich, S.; Goerigk, L. Effect of the Damping Function in Dispersion Corrected Density Functional Theory. *J. Comput. Chem.* **2011,** *32*, 1456-1465.
(14) A. P. Rahalkar; A. Stanger *Aroma, http://schulich.technion.ac.il/Amnon_Stanger.htm* 2014.
(15) Geuenich, D.; Hess, K.; Köhler, F.; Herges, R. Anisotropy of the Induced Current Density (ACID), a General Method To Quantify and Visualize Electronic Delocalization. *Chem. Rev.* **2005,** *105*, 3758-3772.
(16) M. J. Frisch, G. W. Trucks, H. B. Schlegel, G. E. Scuseria, M. A. Robb, J. R. Cheeseman, G. Scalmani, V. Barone, B. Mennucci, G. A. Petersson, H. Nakatsuji, M. Caricato, X. Li, H. P. Hratchian, A. F. Izmaylov, J. Bloino, G. Zheng, J. L. Sonnenberg, M. Hada, M. Ehara, K. Toyota,

R. Fukuda, J. Hasegawa, M. Ishida, T. Nakajima, Y. Honda, O. Kitao, H. Nakai, T. Vreven, J. A. Montgomery, Jr., J. E. Peralta, F. Ogliaro, M. Bearpark, J. J. Heyd, E. Brothers, K. N. Kudin, V. N. Staroverov, R. Kobayashi, J. Normand, K. Raghavachari, A. Rendell, J. C. Burant, S. S. Iyengar, J. Tomasi, M. Cossi, N. Rega, J. M. Millam, M. Klene, J. E. Knox, J. B. Cross, V. Bakken, C. Adamo, J. Jaramillo, R. Gomperts, R. E. Stratmann, O. Yazyev, A. J. Austin, R. Cammi, C. Pomelli, J. W. Ochterski, R. L. Martin, K. Morokuma, V. G. Zakrzewski, G. A. Voth, P. Salvador, J. J. Dannenberg, S. Dapprich, A. D. Daniels, Ö. Farkas, J. B. Foresman, J. V. Ortiz, J. Cioslowski, and D. J. Fox, Gaussian 09, Revision D.01, Gaussian, Inc., Wallingford CT, **2009**.
